# Supplementary material for: Oxidative metabolisms catalyzed Earth’s oxygenation
Source: Nat Commun. 2022 Mar 14;13:1328. doi: 10.1038/s41467-022-28996-0 (PMC8921266; doi:10.1038/s41467-022-28996-0)
Supplement: Supplementary file 1 — Supplementary Information [file 41467_2022_28996_MOESM1_ESM.pdf]

Oxidative metabolisms catalyzed Earth's oxygenation
# – Supplementary Information –

Haitao Shang, [1,2,*] Daniel H. Rothman, [1,2] Gregory P. Fournier [2]

[1]Lorenz Center, [2]Department of Earth, Atmospheric, and Planetary Sciences,
Massachusetts Institute of Technology, Cambridge, MA 02139, USA
[*]To whom correspondence should be addressed; E-mail: htshang.research@gmail.com.

Table **S1**: List of Ribosomal Proteins

Large Subunit (50 S)	Small Subunit (30 S)
L1	S2
L2	S3
L3	S4
L4	S5
L5	S7
L6	S8
L10	S9
L13	S10
L14	S11
L15	S12
L18	S13
L22	S14
L23	S15
L24	S17
L29	S19

Table **S2**: Information of Taxa

Taxon Name	Database	Genome ID
1. *Rickettsia typhi* str. B9991CWPP	PATRIC	1003202.3 [https://www.patricbrc.org/view/Genome/1003202.3]
2. *Parvularcula bermudensis* HTCC2503	PATRIC	314260.8 [https://www.patricbrc.org/view/Genome/314260.8]
3. *Pelagibaca bermudensis* HTCC2601	PATRIC	314265.3 [https://www.patricbrc.org/view/Genome/314265.3]
4. *Rhodospirillum centenum* SW	PATRIC	414684.5 [https://www.patricbrc.org/view/Genome/414684.5]
5. *Methylobacterium radiotolerans* JCM 2831	PATRIC	426355.14 [https://www.patricbrc.org/view/Genome/426355.14]
6. *Caulobacter segnis* ATCC 21756	PATRIC	509190.6 [https://www.patricbrc.org/view/Genome/509190.6]
7. *Candidatus Pelagibacter ubique* HTCC1062	PATRIC	335992.9 [https://www.patricbrc.org/view/Genome/335992.9]
8. *Pedobacter kyungheensis* KACC 16221	PATRIC	1069985.4 [https://www.patricbrc.org/view/Genome/1069985.4]
9. *Blattabacterium* sp. (*Cryptocercus punctulatus*) str. Cpu	PATRIC	1075399.3 [https://www.patricbrc.org/view/Genome/1075399.3]
10. *Formosa* sp. AK20	PATRIC	1137281.3 [https://www.patricbrc.org/view/Genome/1137281.3]
11. *Mangrovimonas yunxiaonensisstr.* LY01	PATRIC	1197477.3 [https://www.patricbrc.org/view/Genome/1197477.3]
12. *Cellulophaga geojensis* KL-A	PATRIC	1328323.3 [https://www.patricbrc.org/view/Genome/1328323.3]
13. *Cardinium endosymbiont* cBtQ1 of Bemisia tabaci	PATRIC	1354314.3 [https://www.patricbrc.org/view/Genome/1354314.3]
14. *Elizabethkingia meningoseptica* 502	PATRIC	1355388.3 [https://www.patricbrc.org/view/Genome/1355388.3]
15. *Rikenellaceae bacterium* M3	PATRIC	1433126.3 [https://www.patricbrc.org/view/Genome/1433126.3]
16. *Flavobacterium aquatile* LMG 4008	PATRIC	1453498.8 [https://www.patricbrc.org/view/Genome/1453498.8]
17. *Schleiferia thermophila* str. Yellowstone	PATRIC	1453500.3 [https://www.patricbrc.org/view/Genome/1453500.3]
18. *Chryseobacterium antarcticum*	PATRIC	266748.6 [https://www.patricbrc.org/view/Genome/266748.6]
19. *Capnocytophaga canimorsus* str. CcD38	PATRIC	28188.6 [https://www.patricbrc.org/view/Genome/28188.6]
20. *Robiginitalea biformata* HTCC2501	PATRIC	313596.4 [https://www.patricbrc.org/view/Genome/313596.4]
21. *Porphyromonas canoris* COT-108$_{O}H$1224	PATRIC	36875.4 [https://www.patricbrc.org/view/Genome/36875.4]
22. *Kordia algicida* OT-1	PATRIC	391587.3 [https://www.patricbrc.org/view/Genome/391587.3]
23. *Epilithonimonas lactis* str. LMG 24401	PATRIC	421072.8 [https://www.patricbrc.org/view/Genome/421072.8]
24. *Chryseobacterium luteum* str. DSM 18605	PATRIC	421531.5 [https://www.patricbrc.org/view/Genome/421531.5]
25. *Parabacteroides distasonis* ATCC 8503	PATRIC	435591.13 [https://www.patricbrc.org/view/Genome/435591.13]
26. *Bacteroides fragilis* 3$_{11}$2	PATRIC	457424.5 [https://www.patricbrc.org/view/Genome/457424.5]
27. *Spirosoma linguale* DSM 74	PATRIC	504472.7 [https://www.patricbrc.org/view/Genome/504472.7]
28. *Nonlabens dokdonensis* DSW-6	PATRIC	592029.3 [https://www.patricbrc.org/view/Genome/592029.3]
29. *Marivirga tractuosa* DSM 4126	PATRIC	643867.3 [https://www.patricbrc.org/view/Genome/643867.3]
30. *Paludibacter propionicigenes* WB4	PATRIC	694427.4 [https://www.patricbrc.org/view/Genome/694427.4]
31. *Paraprevotella clara* YIT 11840	PATRIC	762968.3 [https://www.patricbrc.org/view/Genome/762968.3]
32. *Weeksella virosa* DSM 16922	PATRIC	865938.3 [https://www.patricbrc.org/view/Genome/865938.3]
33. *Ornithobacterium rhinotracheale* DSM 15997	PATRIC	867902.3 [https://www.patricbrc.org/view/Genome/867902.3]
34. *Niabella soli* DSM 19437	PATRIC	929713.3 [https://www.patricbrc.org/view/Genome/929713.3]
35. *Prevotella nigrescens* ATCC 33563	PATRIC	997352.4 [https://www.patricbrc.org/view/Genome/997352.4]
36. *Fluviicola taffensis* DSM 16823	PATRIC	755732.3 [https://www.patricbrc.org/view/Genome/755732.3]
37. *Owenweeksia hongkongensis* DSM 17368	PATRIC	926562.3 [https://www.patricbrc.org/view/Genome/926562.3]
38. *Blattabacterium* sp. (*Blaberus giganteus*)	PATRIC	1186051.3 [https://www.patricbrc.org/view/Genome/1186051.3]
39. *Chlorobium tepidum* TLS	PATRIC	194439.7 [https://www.patricbrc.org/view/Genome/194439.7]
40. *Chlorobium phaeobacteroides* BS1	PATRIC	331678.5 [https://www.patricbrc.org/view/Genome/331678.5]

Continued

Taxon Name	Database	Genome ID
41. *Chlorobium ferrooxidanDSM* 13031	PATRIC	377431.3 [https://www.patricbrc.org/view/Genome/377431.3]
42. *Chlorobium chlorochromatii* CaD3	PATRIC	340177.11 [https://www.patricbrc.org/view/Genome/340177.11]
43. *Chlorobium phaeovibrioides* DSM 265	PATRIC	290318.6 [https://www.patricbrc.org/view/Genome/290318.6]
44. *Chlorobium luteolum* DSM 273	PATRIC	319225.5 [https://www.patricbrc.org/view/Genome/319225.5]
45. *Chlorobium phaeobacteroides* DSM 266	PATRIC	290317.9 [https://www.patricbrc.org/view/Genome/290317.9]
46. *Chlorobaculum parvum* NCIB 8327	PATRIC	517417.5 [https://www.patricbrc.org/view/Genome/517417.5]
47. *Chlorobium limicola* DSM 245	PATRIC	290315.5 [https://www.patricbrc.org/view/Genome/290315.5]
48. *Pelodictyon phaeoclathratiforme* BU-1	PATRIC	324925.5 [https://www.patricbrc.org/view/Genome/324925.5]
49. *Chloroherpeton thalassium* ATCC 35110	PATRIC	517418.5 [https://www.patricbrc.org/view/Genome/517418.5]
50. *Prosthecochloris aestuarii* DSM 271	PATRIC	290512.6 [https://www.patricbrc.org/view/Genome/290512.6]
51. *Nitrolancetus hollandicus* Lb	PATRIC	1129897.3 [https://www.patricbrc.org/view/Genome/1129897.3]
52. *Dehalococcoides mccartyi* CG1	PATRIC	1432059.3 [https://www.patricbrc.org/view/Genome/1432059.3]
53. *Thermomicrobium roseum* DSM 5159	PATRIC	309801.4 [https://www.patricbrc.org/view/Genome/309801.4]
54. *Chloroflexus aurantiacus* J-10-fl	PATRIC	324602.8 [https://www.patricbrc.org/view/Genome/324602.8]
55. *Chloroflexus aggregans* DSM 9485	PATRIC	326427.4 [https://www.patricbrc.org/view/Genome/326427.4]
56. *Sphaerobacter thermophilus* DSM 20745	PATRIC	479434.6 [https://www.patricbrc.org/view/Genome/479434.6]
57. *Ktedonobacter racemifer* DSM 44963	PATRIC	485913.3 [https://www.patricbrc.org/view/Genome/485913.3]
58. *Dehalogenimonas lykanthroporepellens* BL-DC-9	PATRIC	552811.9 [https://www.patricbrc.org/view/Genome/552811.9]
59. *Caldilinea aerophila* DSM 14535 = NBRC 104270	PATRIC	926550.5 [https://www.patricbrc.org/view/Genome/926550.5]
60. *Anaerolinea thermophila* UNI-1	PATRIC	926569.3 [https://www.patricbrc.org/view/Genome/926569.3]
61. *Roseiflexus* sp. RS-1	PATRIC	357808.5 [https://www.patricbrc.org/view/Genome/357808.5]
62. *Roseiflexus castenholzii* DSM 13941	PATRIC	383372.6 [https://www.patricbrc.org/view/Genome/383372.6]
63. *Prosthecochloris aestuarii* DSM 271	PATRIC	290512.6 [https://www.patricbrc.org/view/Genome/290512.6]
64. *Leptolyngbya* sp. PCC 7375	PATRIC	102129.3 [https://www.patricbrc.org/view/Genome/102129.3]
65. *Nostoc* sp. PCC 7120	PATRIC	103690.1 [https://www.patricbrc.org/view/Genome/103690.1]
66. *Stanieria cyanosphaera* PCC 7437	PATRIC	111780.3 [https://www.patricbrc.org/view/Genome/111780.3]
67. *Geitlerinema* sp. PCC 7407	PATRIC	1173025.3 [https://www.patricbrc.org/view/Genome/1173025.3]
68. *Coleofasciculus chthonoplastes* PCC 7420	PATRIC	118168.3 [https://www.patricbrc.org/view/Genome/118168.3]
69. *Prochlorococcus marinus* subsp. marinus str. CCMP1375	PATRIC	167539.5 [https://www.patricbrc.org/view/Genome/167539.5]
70. *Thermosynechococcus elongatus* BP-1	PATRIC	197221.4 [https://www.patricbrc.org/view/Genome/197221.4]
71. *Trichodesmium erythraeum* IMS101	PATRIC	203124.6 [https://www.patricbrc.org/view/Genome/203124.6]
72. *Gloeobacter violaceus* PCC 7421	PATRIC	251221.4 [https://www.patricbrc.org/view/Genome/251221.4]
73. *Chroococcidiopsis thermalis* PCC 7203	PATRIC	251229.3 [https://www.patricbrc.org/view/Genome/251229.3]
74. *Synechococcus elongatus* PCC 6301	PATRIC	269084.6 [https://www.patricbrc.org/view/Genome/269084.6]
75. *Cyanobium gracile* PCC 6307	PATRIC	292564.3 [https://www.patricbrc.org/view/Genome/292564.3]
76. *Rivularia* sp. PCC 7116	PATRIC	373994.3 [https://www.patricbrc.org/view/Genome/373994.3]
77. *Cyanothece* sp. PCC 7822	PATRIC	497965.6 [https://www.patricbrc.org/view/Genome/497965.6]
78. *Arthrospira platensis* NIES-39	PATRIC	696747.3 [https://www.patricbrc.org/view/Genome/696747.3]
79. *Pseudanabaena* sp. PCC 7367	PATRIC	82654.3 [https://www.patricbrc.org/view/Genome/82654.3]
80. *Melioribacter roseus* P3M-2	PATRIC	1191523.3 [https://www.patricbrc.org/view/Genome/1191523.3]
81. *Ignavibacterium album* JCM 16511	PATRIC	945713.3 [https://www.patricbrc.org/view/Genome/945713.3]

Continued

Taxon Name	Database	Genome ID
82. *Obscuribacter phosphatis* (Mle1_12)	IMG	2556921048 [https://genome.jgi.doe.gov/portal/IMG_2556921048]
83. *Gastranaerophilaceae* Zag_1 (Zagget bin 1)	IMG	2523533517 [https://genome.jgi.doe.gov/portal/IMG_2523533517]
84. *Gastranaerophilaceae Zag_111* (Zagget_111_MP)	IMG	2531839741 [https://genome.jgi.doe.gov/portal/IMG_2531839741]
85. *Gastranaerophilus phascolarctosicola* (Zagget bin 221)	IMG	2523533519 [https://genome.jgi.doe.gov/portal/IMG_2523533519]
86. *Sericytochromatia*, from Coal Bed ML635J-21	R. Soo [1]	CBMW_12 [https://www.science.org/doi/10.1126/science.aal3794]
87. *Sericytochromatia*, from Rifle Acetate Amendment	R. Soo [1]	CBMW_12 [https://www.science.org/doi/10.1126/science.aal3794]
88. *Sericytochromatia*, from Photoreactor Bin 72	R. Soo [1]	LSPB_72 [https://www.science.org/doi/10.1126/science.aal3794]
89. *Oscillochlorois trichoides*	NCBI	GCF_000152145.1_ASM15214v1 [https://www.ncbi.nlm.nih.gov/assembly/GCF_000152145]
90. *Dictyobacter aurantiacus*	NCBI	GCA_003967515.1_ASM396751v1 [https://www.ncbi.nlm.nih.gov/assembly/GCF_003967515]
91. *Thermosporothrix hazakensis*	NCBI	GCA_003253565.1_ASM325356v1 [https://www.ncbi.nlm.nih.gov/assembly/GCF_003253565]
92. *Ktedonobacter* sp. 13_2_20CM_53_11	NCBI	GCA_001914715.1_ASM191471v1 [https://www.ncbi.nlm.nih.gov/assembly/GCA_001914715]
93. *Thermogemmatispora carboxidivorans*	NCBI	GCA_000702505.1_ASM70250v1 [https://www.ncbi.nlm.nih.gov/assembly/GCA_000702505]
94. *Thermogemmatispora onikobensis*	NCBI	GCA_001748285.1_ASM174828v1 [https://www.ncbi.nlm.nih.gov/assembly/GCA_001748285]
95. *Thermogemmatispora tikiterensis*	NCBI	GCA_003268475.1_ASM326847v1 [https://www.ncbi.nlm.nih.gov/assembly/GCA_003268475]
96. *Kouleothrix aurantiaca*	NCBI	GCA_001399705.1_ASM139970v1 [https://www.ncbi.nlm.nih.gov/assembly/GCA_001399705]
97. *Candidatus Chloroploca asiatica*	NCBI	GCA_002532075.1_ASM253207v1 [https://www.ncbi.nlm.nih.gov/assembly/GCA_002532075]
98. *Candidatus Viridilinea halotolerans*	NCBI	GCA_003934145.1_ASM393414v1 [https://www.ncbi.nlm.nih.gov/assembly/GCA_003934145]
99. *Chloroflexus islandicus*	NCBI	GCA_001650695.1_ASM165069v1 [https://www.ncbi.nlm.nih.gov/assembly/GCA_001650695]
100. *Herpetosiphon geysericola*	NCBI	GCA_001306135.1_ASM130613v1 [https://www.ncbi.nlm.nih.gov/assembly/GCA_001306135]
101. *Herpetosiphon llansteffanense*	NCBI	GCA_003205875.1_ASM320587v1 [https://www.ncbi.nlm.nih.gov/assembly/GCA_003205875]
102. *Thermorudis peleae*	NCBI	GCA_000744775.1_ASM74477v1 [https://www.ncbi.nlm.nih.gov/assembly/GCA_000744775]
103. *Litorilinea aerophila*	NCBI	GCA_002148365.1_ASM214836v1 [https://www.ncbi.nlm.nih.gov/assembly/GCA_002148365]
104. *Ardenticatena maritima*	NCBI	GCA_001293545.1_ASM129354v1 [https://www.ncbi.nlm.nih.gov/assembly/GCA_001293545]
105. *Ardenticatenia bacterium*	NCBI	GCA_003130875.1_ASM313087v1 [https://www.ncbi.nlm.nih.gov/assembly/GCA_003130875]
106. *Thermoflexia bacterium*	NCBI	GCA_003694475.1_ASM369447v1 [https://www.ncbi.nlm.nih.gov/assembly/GCA_003694475]
107. *Anaerolineae bacterium* SG8_19	NCBI	GCA_001303105.1_ASM130310v1 [https://www.ncbi.nlm.nih.gov/assembly/GCA_001303105]
108. *Candidatus Promineofilum breve*	NCBI	GCA_900066015.1_Cfx-K [https://www.ncbi.nlm.nih.gov/assembly/GCA_900066015]
109. *Anaerolineaebacterium* SM23_63	NCBI	GCA_001303965.1_ASM130396v1 [https://www.ncbi.nlm.nih.gov/assembly/GCA_001303965]
110. *Brevefilum fermentans*	NCBI	GCA_900184705.1_CAMBI-1 [https://www.ncbi.nlm.nih.gov/assembly/GCA_900184705]
111. *Anaerolineae bacterium* UTCFX2	NCBI	GCA_002050125.1_ASM205012v1 [https://www.ncbi.nlm.nih.gov/assembly/GCA_002050125]
112. *Flexilinea flocculi*	NCBI	GCA_001192795.1_ASM119279v1 [https://www.ncbi.nlm.nih.gov/assembly/GCA_001192795]
113. *Levilinea saccharolytica*	NCBI	GCA_001306035.1_ASM130603v1 [https://www.ncbi.nlm.nih.gov/assembly/GCA_001306035]
114. *Ornatilinea apprima*	NCBI	GCA_001306115.1_ASM130611v1 [https://www.ncbi.nlm.nih.gov/assembly/GCA_001306115]
115. *Longilinea arvoryzae*	NCBI	GCA_001050235.2_ASM105023v2 [https://www.ncbi.nlm.nih.gov/assembly/GCA_001050235]
116. *Leptolinea tardivitalis*	NCBI	GCA_001050275.1_ASM105027v1 [https://www.ncbi.nlm.nih.gov/assembly/GCA_001050275]
117. *Thermanaerothrix daxensis*	NCBI	GCA_001306145.1_ASM130614v1 [https://www.ncbi.nlm.nih.gov/assembly/GCA_001306145]
118. *Bellilinea caldifistulae*	NCBI	GCA_001306055.1_ASM130605v1 [https://www.ncbi.nlm.nih.gov/assembly/GCA_001306055]
119. *Pelolinea submarina*	NCBI	GCA_003966975.1_ASM396697v1 [https://www.ncbi.nlm.nih.gov/assembly/GCA_003966975]
120. *Anaerolinea* sp. 4484_236	NCBI	GCA_002085065.1_ASM208506v1 [https://www.ncbi.nlm.nih.gov/assembly/GCA_002085065]
121. *Anaerolineae bacterium* CG2_30_58_95	NCBI	GCA_001872165.1_ASM187216v1 [https://www.ncbi.nlm.nih.gov/assembly/GCA_001872165]

Continued

Taxon Name	Database	Genome ID
122. *SAR202 cluster bacterium* Io17-Chloro-G1	NCBI	GCA_002816375.1_ ASM281637v1 [https://www.ncbi.nlm.nih.gov/assembly/GCA_002816375]
123. *SAR202 cluster bacterium* Io17-Chloro-G2	NCBI	GCA_002816875.1_ASM281687v1 [https://www.ncbi.nlm.nih.gov/assembly/GCA_002816875]
124. *SAR202 cluster bacterium* Io17-Chloro-G3	NCBI	GCA_002816985.1_ ASM281698v1 [https://www.ncbi.nlm.nih.gov/assembly/GCA_002816985]
125. *SAR202 cluster bacterium* Io17-Chloro-G4	NCBI	GCA_002816455.1_ ASM281645v1 [https://www.ncbi.nlm.nih.gov/assembly/GCA_002816455]
126. *SAR202 cluster bacterium* Io17-Chloro-G5	NCBI	GCA_002816355.1_ ASM281635v1 [https://www.ncbi.nlm.nih.gov/assembly/GCA_002816355]
127. *SAR202 cluster bacterium* Io17-Chloro-G6	NCBI	GCA_002816585.1_ ASM281658v1 [https://www.ncbi.nlm.nih.gov/assembly/GCA_002816585]
128. *SAR202 cluster bacterium* Io17-Chloro-G7	NCBI	GCA_002817055.1_ ASM281705v1 [https://www.ncbi.nlm.nih.gov/assembly/GCA_002817055]
129. *SAR202 cluster bacterium* Io17-Chloro-G8	NCBI	GCA_002817135.1_ ASM281713v1 [https://www.ncbi.nlm.nih.gov/assembly/GCA_002817135]
130. *SAR202 cluster bacterium* Io17-Chloro-G9	NCBI	GCA_002816675.1_ ASM281667v1 [https://www.ncbi.nlm.nih.gov/assembly/GCA_002816675]
131. *SAR202 cluster bacterium* Casp-Chloro-G2	NCBI	GCA_002816575.1_ ASM281657v1 [https://www.ncbi.nlm.nih.gov/assembly/GCA_002816575]
132. *SAR202 cluster bacterium* Casp-Chloro-G3	NCBI	GCA_002816715.1_ ASM281671v1 [https://www.ncbi.nlm.nih.gov/assembly/GCA_002816715]
133. *SAR202 cluster bacterium* Casp-Chloro-G4	NCBI	GCA_002816705.1_ ASM281670v1 [https://www.ncbi.nlm.nih.gov/assembly/GCA_002816705]
134. *SAR202 cluster bacterium* Ae2-Chloro-G2	NCBI	GCA_002816925.1_ ASM281692v1 [https://www.ncbi.nlm.nih.gov/assembly/GCA_002816925]
135. *SAR202 cluster bacterium* MP-SInd-SRR3963457-G1	NCBI	GCA_002817235.1_ ASM281723v1 [https://www.ncbi.nlm.nih.gov/assembly/GCA_002817235]
136. *SAR202 cluster bacterium* MP-SInd-SRR3963457-G2	NCBI	GCA_002816735.1_ ASM281673v1 [https://www.ncbi.nlm.nih.gov/assembly/GCA_002816735]
137. *SAR202 cluster bacterium* MP-NPac-SRR3961935-G1	NCBI	GCA_002817285.1_ ASM281728v1 [https://www.ncbi.nlm.nih.gov/assembly/GCA_002817285]
138. *SAR202 cluster bacterium* MP-SAtl-SRR3965592-G1	NCBI	GCA_002817125.1_ ASM281712v1 [https://www.ncbi.nlm.nih.gov/assembly/GCA_002817125]
139. *Fischerella* sp. JSC-11	NCBI	GCF_000231365.1_ASM23136v1 [https://www.ncbi.nlm.nih.gov/assembly/GCA_000231365]
140. *Prochlorothrix hollandicaPCC* 9006 = CALU 1027	NCBI	GCF_000332315.1_ ASM33231v1 [https://www.ncbi.nlm.nih.gov/assembly/GCA_000332315]
141. *Wolbachia endosymbiont of Drosophila simulans* wNo	NCBI	GCA_000376585.1_ ASM37658v1 [https://www.ncbi.nlm.nih.gov/assembly/GCA_000376585]
142. *Anaplasma phagocytophilum* str. Webster	NCBI	GCA_000964685.1_ ASM96468v1 [https://www.ncbi.nlm.nih.gov/assembly/GCA_000964685]
143. *Magnetococcus marinus* MC-1	NCBI	GCA_000014865.1_ASM1486v1 [https://www.ncbi.nlm.nih.gov/assembly/GCA_000014865]
144. *Ehrlichia canis* str. Jake	NCBI	GCA_000012565.1_ASM1256v1 [https://www.ncbi.nlm.nih.gov/assembly/GCA_000012565]
145. *Gemmatimonadetes bacterium*	NCBI	GCA_003134685.1_20111000_P3M [https://www.ncbi.nlm.nih.gov/assembly/GCA_003134685]
146. *Candidatus Entotheonella factor*	NCBI	GCA_000522425.1v3 [https://www.ncbi.nlm.nih.gov/assembly/GCA_000522425]
147. *Candidatus Entotheonella* sp. TSY2	NCBI	GCA_000522445.1v3 [https://www.ncbi.nlm.nih.gov/assembly/GCA_000522445]
148. *Candidatus Entotheonella palauensis*	NCBI	GCA_900079095.1_E.gemina.ts [https://www.ncbi.nlm.nih.gov/assembly/GCA_900079095]
149. *Candidatus Entotheonella serta*	NCBI	GCA_002914425.1_ASM291442v1 [https://www.ncbi.nlm.nih.gov/assembly/GCA_002914425]
150. *Candidatus Rokubacteria bacterium* 13_1_40CM_2_68_8	NCBI	GCA_001919035.1_ASM191903v1 [https://www.ncbi.nlm.nih.gov/assembly/GCA_001919035]
151. *Rhodospirillales bacterium* 35-66-84	NCBI	GCA_002281325.1_ASM228132v1 [https://www.ncbi.nlm.nih.gov/assembly/GCA_002281325]
152. *Reyranella* sp.	NCBI	GCA_004297405.1_ASM429740v1 [https://www.ncbi.nlm.nih.gov/assembly/GCA_004297405]
153. *Reyranella massiliensis*	NCBI	GCA_000312425.1_ASM31242v1 [https://www.ncbi.nlm.nih.gov/assembly/GCA_000312425]
154. *Enhydrobacter aerosaccus*	NCBI	GCA_900167455.1_IMG-taxon [https://www.ncbi.nlm.nih.gov/assembly/GCA_900167455]
155. *Alphaproteobacteria bacterium* 65-37	NCBI	GCA_001897995.1_ASM189799v1 [https://www.ncbi.nlm.nih.gov/assembly/GCA_001897995]
156. *Rhodospirillaceae bacterium* CCH5-H10	NCBI	GCA_001557035.1_ASM155703v1 [https://www.ncbi.nlm.nih.gov/assembly/GCA_001557035]
157. *Rhodospirillales bacterium* 69-11	NCBI	GCA_001898425.1_ASM189842v1 [https://www.ncbi.nlm.nih.gov/assembly/GCA_001898425]
158. *Belnapia moabensis*	NCBI	GCA_000745835.1_ASM74583v1 [https://www.ncbi.nlm.nih.gov/assembly/GCA_000745835]
159. *Belnapia* sp. F-4-1	NCBI	GCA_000802185.1_ASM80218v1 [https://www.ncbi.nlm.nih.gov/assembly/GCA_000802185]
160. *Rhodospirillaceae bacterium* SYSU D60015	NCBI	GCA_003576705.1_ASM357670v1 [https://www.ncbi.nlm.nih.gov/assembly/GCA_003576705]
161. *Thermoflexus hugenholtzii*	NCBI	GCA_002563855.1_ASM256385v1 [https://www.ncbi.nlm.nih.gov/assembly/GCA_002563855]

Continued

Taxon Name	Database	Genome ID
162. *Rhodospirillales bacterium* URHD0017	NCBI	GCA_900110395.1_IMG-taxon [https://www.ncbi.nlm.nih.gov/assembly/GCA_900110395]
163. *Humitalea rosea*	NCBI	GCA_003253705.1_ASM325370v1 [https://www.ncbi.nlm.nih.gov/assembly/GCA_003253705]
164. *Roseomonas stagni*	NCBI	GCA_900114315.1_IMG-taxon [https://www.ncbi.nlm.nih.gov/assembly/GCA_900114315]
165. *Rhodospirillales bacterium* 70-18	NCBI	GCA_001899585.1_ASM189958v1 [https://www.ncbi.nlm.nih.gov/assembly/GCA_001899585]
166. *Paracraurococcus ruber*	NCBI	GCA_004353985.1_ASM435398v1 [https://www.ncbi.nlm.nih.gov/assembly/GCA_004353985]
167. *beta proteobacterium MWH-P2sevCIIIb*	NCBI	GCA_003003055.1_ASM300305v1 [https://www.ncbi.nlm.nih.gov/assembly/GCA_003003055]
168. *Rhodospirillales bacterium* 69-11	NCBI	GCA_001898425.1_ASM189842v1 [https://www.ncbi.nlm.nih.gov/assembly/GCA_001898425]
169. *Rhodospirillaceae bacterium* SYSU D60007	NCBI	GCA_003574685.1_ASM357468v1 [https://www.ncbi.nlm.nih.gov/assembly/GCA_003574685]
170. *Hyphomicrobium* sp. CS1BSMeth3	NCBI	GCA_900117415.1_CS1BSMeth3 [https://www.ncbi.nlm.nih.gov/assembly/GCA_900117415]
171. *Streptomyces* sp. Ag109_O5-10	NCBI	GCA_900105755.1_IMG-taxon [https://www.ncbi.nlm.nih.gov/assembly/GCA_900105755]
172. *Acetobacteraceae bacterium*	NCBI	GCA_004843345.1_ASM484334v1 [https://www.ncbi.nlm.nih.gov/assembly/GCA_004843345]
173. *Dankookia rubra*	NCBI	GCA_004355005.1_ASM435500v1 [https://www.ncbi.nlm.nih.gov/assembly/GCA_004355005]
174. *Paracraurococcus* sp. NE82	NCBI	GCA_004343615.1_ASM434361v1 [https://www.ncbi.nlm.nih.gov/assembly/GCA_004343615]
175. *Streptomyces fulvoviolaceus*	NCBI	GCA_000718165.1_ASM71816v1 [https://www.ncbi.nlm.nih.gov/assembly/GCA_000718165]
176. *Tepidicaulis* sp. EA10	NCBI	GCA_003688365.1_ASM368836v1 [https://www.ncbi.nlm.nih.gov/assembly/GCA_003688365]
177. *Roseococcus* sp. SYP-B2431	NCBI	GCA_004336745.1_ASM433674v1 [https://www.ncbi.nlm.nih.gov/assembly/GCA_004336745]
178. *Streptomyces mirabilis*	NCBI	GCA_000746395.1_ASM74639v1 [https://www.ncbi.nlm.nih.gov/assembly/GCA_00074639]
179. *Streptomyces* sp. SA15	NCBI	GCA_002291145.1_ASM229114v1 [https://www.ncbi.nlm.nih.gov/assembly/GCA_002291145]
180. *Cystobacter fuscus*	NCBI	GCA_002305875.1_ASM230587v1 [https://www.ncbi.nlm.nih.gov/assembly/GCA_002305875]
181. *Streptomyces albus*	NCBI	GCA_000827005.1_ASM82700v1 [https://www.ncbi.nlm.nih.gov/assembly/GCA_000827005]
182. *Mycobacterium* sp. AB57	NCBI	GCA_900157375.1_PRJEB19165 [https://www.ncbi.nlm.nih.gov/assembly/GCA_900157375]
183. *Streptomyces puniciscabiei*	NCBI	GCA_001735805.1_ASM173580v1 [https://www.ncbi.nlm.nih.gov/assembly/GCA_001735805]
184. *Alphaproteobacteria bacterium* HGW-Alphaproteobacteria-3	NCBI	GCA_002842875.1_ASM284287v1 [https://www.ncbi.nlm.nih.gov/assembly/GCA_002842875]
185. *Frankia* sp. EUN1f	NCBI	GCA_000177675.1_ASM17767v1 [https://www.ncbi.nlm.nih.gov/assembly/GCA_000177675]
186. *Parvibaculum lavamentivorans*	NCBI	GCA_000017565.1_ASM1756v1 [https://www.ncbi.nlm.nih.gov/assembly/GCA_000017565]
187. *Mycolicibacterium moriokaense*	NCBI	GCA_002086395.1_ASM208639v1 [https://www.ncbi.nlm.nih.gov/assembly/GCA_002086395]
188. *Acidimicrobiaceae bacterium*	NCBI	GCA_002694625.1_ASM269462v1 [https://www.ncbi.nlm.nih.gov/assembly/GCA_002694625]
189. *Phenylobacterium* sp. LX32	NCBI	GCA_003254475.1_ASM325447v1 [https://www.ncbi.nlm.nih.gov/assembly/GCA_003254475]
190. *Streptomyces* sp. SM1	NCBI	GCA_002910825.1_ASM291082v1 [https://www.ncbi.nlm.nih.gov/assembly/GCA_002910825]
191, *Streptomyces rimosus*	NCBI	GCA_004196335.1_ASM419633v1 [https://www.ncbi.nlm.nih.gov/assembly/GCA_004196335]
192. *Alphaproteobacteria bacterium* HGW-Alphaproteobacteria-11	NCBI	GCA_002841195.1_ASM284119v1 [https://www.ncbi.nlm.nih.gov/assembly/GCA_002841195]
193. *Euzebya tangerina*	NCBI	GCA_003074135.1_ASM307413v1 [https://www.ncbi.nlm.nih.gov/assembly/GCA_003074135]
194. *Ilumatobacter fluminis*	NCBI	GCA_004364865.1_ASM436486v1 [https://www.ncbi.nlm.nih.gov/assembly/GCA_004364865]
195. *Rhodosalinus* sp. E84	NCBI	GCA_003298775.1_ASM329877v1 [https://www.ncbi.nlm.nih.gov/assembly/GCA_003298775]
196. *Mycobacterium palustre*	NCBI	GCA_002101785.1_ASM210178v1 [https://www.ncbi.nlm.nih.gov/assembly/GCA_002101785]
197. *Ilumatobacter nonamiensis*	NCBI	GCA_000350145.1_ASM35014v1 [https://www.ncbi.nlm.nih.gov/assembly/GCA_000350145]
198. *Mycobacterium* sp. 1274761.0	NCBI	GCA_001668615.1_SM166861v1 [https://www.ncbi.nlm.nih.gov/assembly/GCA_001668615]
199. *Mycobacterium triplex*	NCBI	GCA_002102415.1_ASM210241v1 [https://www.ncbi.nlm.nih.gov/assembly/GCA_002102415]
200. *Caulobacteraceae bacterium*	NCBI	GCA_003164925.1_20120700_S1D [https://www.ncbi.nlm.nih.gov/assembly/GCA_003164925]
201. *Sphingomonas mucosissima*	NCBI	GCA_002197665.1_ASM219766v1 [https://www.ncbi.nlm.nih.gov/assembly/GCA_002197665]
202. *Salinispora pacifica*	NCBI	GCA_000514775.1_ASM51477v1 [https://www.ncbi.nlm.nih.gov/assembly/GCA_000514775]

Continued

Taxon Name	Database	Genome ID
203. *Caulobacter mirabilis*	NCBI	GCA_002749615.1_ASM274961v1 [https://www.ncbi.nlm.nih.gov/assembly/GCA_002749615]
204. *Rubritepida flocculans*	NCBI	GCA_000425365.1_ASM42536v1 [https://www.ncbi.nlm.nih.gov/assembly/GCA_000425365]
205. *Phenylobacterium* sp. SCN 70-31	NCBI	GCA_001724605.1_ASM172460v1 [https://www.ncbi.nlm.nih.gov/assembly/GCA_001724605]
206. *Sphingomonas* sp. 66-10	NCBI	GCA_001897045.1_ASM189704v1 [https://www.ncbi.nlm.nih.gov/assembly/GCA_001897045]
207. *Phenylobacterium* sp. RIFCSPHIGHO2_01_FULL_69_31	NCBI	GCA_001824475.1_ASM182447v1 [https://www.ncbi.nlm.nih.gov/assembly/GCA_001824475]
208. *Mycobacterium* sp. 1100029.7	NCBI	GCA_001665235.1_ASM166523v1 [https://www.ncbi.nlm.nih.gov/assembly/GCA_001665235]
209. *Sphingomonas* sp. 67-36	NCBI	GCA_001897375.1_ASM189737v1 [https://www.ncbi.nlm.nih.gov/assembly/GCA_001897375]
210. *Cryptosporangium aurantiacum*	NCBI	GCA_900143005.1_IMG-taxon [https://www.ncbi.nlm.nih.gov/assembly/GCA_900143005]
211. *Phenylobacterium zucineum*	NCBI	GCA_c.1_ASM324335v1 [https://www.ncbi.nlm.nih.gov/assembly/GCA_900143005]
212. *Sphingomonas jeddahensis*	NCBI	GCA_001981525.1_ASM198152v1 [https://www.ncbi.nlm.nih.gov/assembly/GCA_001981525]
213. *Frankia asymbiotica*	NCBI	GCA_001983105.1_ASM198310v1 [https://www.ncbi.nlm.nih.gov/assembly/GCA_001983105]
214. *Mycobacterium arosiense*	NCBI	GCA_002086125.1_ASM208612v1 [https://www.ncbi.nlm.nih.gov/assembly/GCA_002086125]
215. *Mycobacterium mantenii*	NCBI	GCA_002086335.1_ASM208633v1 [https://www.ncbi.nlm.nih.gov/assembly/GCA_002086335]
216. *Mycobacterium marseillense*	NCBI	GCA_002285715.1_ASM228571v1 [https://www.ncbi.nlm.nih.gov/assembly/GCA_002285715]
217. *Streptomyces scabrisporus*	NCBI	GCA_000372745.1_ASM37274v1 [https://www.ncbi.nlm.nih.gov/assembly/GCA_000372745]
218. *Mycobacterium conspicuum*	NCBI	GCA_002102095.1_ASM210209v1 [https://www.ncbi.nlm.nih.gov/assembly/GCA_002102095]
219. *Phenylobacterium* sp. Root700	NCBI	GCA_001429025.1_Root700 [https://www.ncbi.nlm.nih.gov/assembly/GCA_001429025]
220. *Actinobacteria bacterium* IMCC26207	NCBI	GCA_001025035.1_ASM102503v1 [https://www.ncbi.nlm.nih.gov/assembly/GCA_001025035]
221. *Verrucosispora* sp. SN26$_1$4.1	NCBI	GCA_004307965.1_ASM430796v1 [https://www.ncbi.nlm.nih.gov/assembly/GCA_004307965]
222. *Sphingomonas* sp. Leaf412	NCBI	GCA_001425405.1_Leaf412 [https://www.ncbi.nlm.nih.gov/assembly/GCA_001425405]
223. *Mycobacterium* sp. EPG1	NCBI	GCA_002946335.1_ASM294633v1 [https://www.ncbi.nlm.nih.gov/assembly/GCA_002946335]
224. *Mycolicibacterium chubuense*	NCBI	GCA_000266905.1_ASM26690v1 [https://www.ncbi.nlm.nih.gov/assembly/GCA_000266905]
225. *Chromatiales bacterium* (*Bugula neritina* AB1)	NCBI	GCA_002729495.1_ ASM272949v1 [https://www.ncbi.nlm.nih.gov/assembly/GCA_002729495]
226. *Mycobacterium* sp. NAZ190054	NCBI	GCA_001545925.1_ASM154592v1 [https://www.ncbi.nlm.nih.gov/assembly/GCA_001545925]
227. *Amycolatopsis circi*	NCBI	GCA_003385235.1_ASM338523v1 [https://www.ncbi.nlm.nih.gov/assembly/GCA_003385235]
228. *Mycolicibacterium duvalii*	NCBI	GCA_002553585.1_ASM255358v1 [https://www.ncbi.nlm.nih.gov/assembly/GCA_002553585]
229. *Gemmobacter intermedius*	NCBI	GCA_004054105.1_ASM405410v1 [https://www.ncbi.nlm.nih.gov/assembly/GCA_004054105]
230. *Mycobacterium* sp. AT1	NCBI	GCA_002043095.1_ASM204309v1 [https://www.ncbi.nlm.nih.gov/assembly/GCA_002043095]
231. *Sphingomonas* sp. JJ-A5 (*Tardibacter chloracetimidivorans*)	NCBI	GCA_001890385.1_ASM189038v1 [https://www.ncbi.nlm.nih.gov/assembly/GCA_001890385]
232. *Mycobacterium* sp. Soil538	NCBI	GCA_001428285.1_Soil538 [https://www.ncbi.nlm.nih.gov/assembly/GCA_001428285]
233. *Thalassococcus* sp. S3	NCBI	GCA_004216475.1_ASM421647v1 [https://www.ncbi.nlm.nih.gov/assembly/GCA_004216475]
234. *Mycobacterium* sp. 852013-51886_SCH5428379	NCBI	GCA_001665575.1_ ASM166557v1 [https://www.ncbi.nlm.nih.gov/assembly/GCA_001665575]
235. *Gemmobacter* sp. YIM 102744-1	NCBI	GCA_003863335.1_ASM386333v1 [https://www.ncbi.nlm.nih.gov/assembly/GCA_003863335]
236. *Mycobacterium* sp. Root135	NCBI	GCA_001426545.1_Root135 [https://www.ncbi.nlm.nih.gov/assembly/GCA_001426545]
237. *Candidatus Rhodobacter lobularis*	NCBI	GCA_001078595.1_ASM107859v1 [https://www.ncbi.nlm.nih.gov/assembly/GCA_001078595]
238. *Sphingomonas* sp. TZW2008	NCBI	GCA_002117915.1_SpTZW2008_1.0 [https://www.ncbi.nlm.nih.gov/assembly/GCA_002117915]
239. *Geodermatophilus* sp. Leaf369	NCBI	GCA_001424455.1_Leaf369 [https://www.ncbi.nlm.nih.gov/assembly/GCA_001424455]
240. *Ktedonobacterales bacterium Uno16*	NCBI	GCA_003967575.1_ASM396757v1 [https://www.ncbi.nlm.nih.gov/assembly/GCA_003967575]
241. *Ktedonobacterales bacterium Uno11*	NCBI	GCA_003967555.1_ASM396755v1 [https://www.ncbi.nlm.nih.gov/assembly/GCA_003967555]
242. *Bradyrhizobium* sp. th.b2	NCBI	GCA_000426785.1_ASM42678v1 [https://www.ncbi.nlm.nih.gov/assembly/GCA_000426785]
243. *Nostoc* sp. 3335mG	NCBI	GCA_003185865.1_ASM318586v1 [https://www.ncbi.nlm.nih.gov/assembly/GCA_003185865]

Continued

Taxon Name	Database	Genome ID
244. *Mycobacterium* sp. CECT 8778	NCBI	GCA_002591975.1_ASM259197v1 [https://www.ncbi.nlm.nih.gov/assembly/GCA_002591975]
245. *Ktedonobacterales bacterium* Uno3	NCBI	GCA_003967535.1_ASM396753v1 [https://www.ncbi.nlm.nih.gov/assembly/GCA_003967535]
246. *Dehalococcoidia bacterium* SG8_51_3	NCBI	GCA_001303565.1_ASM130356v1 [https://www.ncbi.nlm.nih.gov/assembly/GCA_001303565]
247. *Dehalococcoidia bacterium* SM23_28_2	NCBI	GCA_001303545.1_ASM130354v1 [https://www.ncbi.nlm.nih.gov/assembly/GCA_001303545]
248. *Dehalococcoidia bacterium* CG2_30_46_9	NCBI	GCA_001873005.1_ ASM187300v1 [https://www.ncbi.nlm.nih.gov/assembly/GCA_]
249. Dehalogenimonas alkenigignens	NCBI	GCA_003095415.1_ASM309541v1 [https://www.ncbi.nlm.nih.gov/assembly/GCA_003095415]
250. *Mycolicibacterium iranicum*	NCBI	GCA_002101705.1_ASM210170v1 [https://www.ncbi.nlm.nih.gov/assembly/GCA_002101705]
251. *Salinispora arenicola* CNS-205 (*Micromonosporaceae*)	NCBI	GCA_000018265.1_ASM1826v1 [https://www.ncbi.nlm.nih.gov/assembly/GCA_000018265]
252. *Gordonia* sp.	NCBI	GCA_003987705.1_ASM398770v1 [https://www.ncbi.nlm.nih.gov/assembly/GCA_003987705]
253. *Albimonas pacifica*	NCBI	GCA_900113695.1_IMG-taxon [https://www.ncbi.nlm.nih.gov/assembly/GCA_900113695]
254. *Alphaproteobacteria bacterium* PA2	NCBI	GCA_002256425.1_ASM225642v1 [https://www.ncbi.nlm.nih.gov/assembly/GCA_002256425]
255. *Streptomycetaceae*	NCBI	GCA_000331185.1_ASM33118v1 [https://www.ncbi.nlm.nih.gov/assembly/GCA_000331185]
256. *Saccharopolyspora flava*	NCBI	GCA_900116135.1_IMG-taxon [https://www.ncbi.nlm.nih.gov/assembly/GCA_900116135]
257. *Bradyrhizobium japonicum*	NCBI	GCA_001887695.1_ASM188769v1 [https://www.ncbi.nlm.nih.gov/assembly/GCA_001887695]
258. *Mycobacterium genavense*	NCBI	GCA_000526915.1_ASM52691v1 [https://www.ncbi.nlm.nih.gov/assembly/GCA_000526915]
259. *Bradyrhizobium* sp. BTAi1	NCBI	GCA_000015165.1_ASM1516v1 [https://www.ncbi.nlm.nih.gov/assembly/GCA_000015165]
260. *Dehalogenimonas formicexedens*	NCBI	GCA_001953175.1_ASM195317v1 [https://www.ncbi.nlm.nih.gov/assembly/GCA_001953175]
261. *Mycolicibacterium rhodesiae*	NCBI	GCA_002086695.1_ASM208669v1 [https://www.ncbi.nlm.nih.gov/assembly/GCA_002086695]
262. *Bradyrhizobium elkanii*	NCBI	GCA_001718185.1_ASM171818v1 [https://www.ncbi.nlm.nih.gov/assembly/GCA_001718185]
263. *Bradyrhizobium* sp. STM 3843	NCBI	GCA_000239815.2_ASM23981v2 [https://www.ncbi.nlm.nih.gov/assembly/GCA_000239815]
264. *Amycolatopsis australiensis*	NCBI	GCA_900119165.1_IMG-taxon [https://www.ncbi.nlm.nih.gov/assembly/GCA_900119165]
265. *Bradyrhizobium valentinum*	NCBI	GCA_001440405.1_ASM144040v1 [https://www.ncbi.nlm.nih.gov/assembly/GCA_001440405]
266. *Bradyrhizobium* sp. Tv2a-2	NCBI	GCA_000472425.1_ASM47242v1 [https://www.ncbi.nlm.nih.gov/assembly/GCA_000472425]
267. *Saccharopolyspora shandongensis*	NCBI	GCA_900106995.1_IMG-taxon [https://www.ncbi.nlm.nih.gov/assembly/GCA_900106995]
268. *Pseudonocardia* sp. CNS-004	NCBI	GCA_001942185.1_ASM194218v1 [https://www.ncbi.nlm.nih.gov/assembly/GCA_001942185]
269. *Acidimicrobiales bacterium* mtb01	NCBI	GCA_004379135.1_ASM437913v1 [https://www.ncbi.nlm.nih.gov/assembly/GCA_004379135]
270. *Minwuia thermotolerans*	NCBI	GCA_002924445.1_ASM292444v1 [https://www.ncbi.nlm.nih.gov/assembly/GCA_002924445]
271. *Parahaliea mediterranea*	NCBI	GCA_003402235.1_ASM340223v1 [https://www.ncbi.nlm.nih.gov/assembly/GCA_003402235]
272. *Rhizobium* sp. BK251	NCBI	GCA_004345245.1_ASM434524v1 [https://www.ncbi.nlm.nih.gov/assembly/GCA_004345245]
273. *Rhizobium Actinomycetospora succinea*	NCBI	GCA_004363095.1_ASM436309v1 [https://www.ncbi.nlm.nih.gov/assembly/GCA_004363095]
274. *Saccharopolyspora* sp. 7K502	NCBI	GCA_004348985.1_ASM434898v1 [https://www.ncbi.nlm.nih.gov/assembly/GCA_004348985]
275. *Nocardia otitidiscaviarum*	NCBI	GCA_900454305.1_54984_D01 [https://www.ncbi.nlm.nih.gov/assembly/GCA_900454305]
276. *Alphaproteobacteria bacterium* 62-8	NCBI	GCA_001898015.1_ASM189801v1 [https://www.ncbi.nlm.nih.gov/assembly/GCA_001898015]
277. *Cumulibacter* sp. G-1	NCBI	GCA_004382795.1_ASM438279v1 [https://www.ncbi.nlm.nih.gov/assembly/GCA_004382795]
278. *Gordonia westfalica*	NCBI	GCA_900105725.1_IMG-taxon [https://www.ncbi.nlm.nih.gov/assembly/GCA_900105725]
279. *Pseudonocardia acaciae*	NCBI	GCA_000620785.1_ASM62078v1 [https://www.ncbi.nlm.nih.gov/assembly/GCA_000620785]
280. *Actinopolyspora erythraea*	NCBI	GCA_002263515.1_ASM226351v1 [https://www.ncbi.nlm.nih.gov/assembly/GCA_002263515]
281. *Sphingomonas* sp. SRS2	NCBI	GCA_000971055.1_SRS2_1.0 [https://www.ncbi.nlm.nih.gov/assembly/GCA_000971055]
282. *Saccharothrix espanaensis*	NCBI	GCA_000328705.1_ASM32870v1 [https://www.ncbi.nlm.nih.gov/assembly/GCA_000328705]
283. *Rhodopila globiformis*	NCBI	GCA_002937115.1_ASM293711v1 [https://www.ncbi.nlm.nih.gov/assembly/GCA_002937115]
284. *Afipia* sp. P52-10	NCBI	GCA_000516555.1_P52-10 [https://www.ncbi.nlm.nih.gov/assembly/GCA_000516555]

Continued

Taxon Name	Database	Genome ID
285. *Afipia broomeae*	NCBI	GCA_002797635.1_ASM279763v1 [https://www.ncbi.nlm.nih.gov/assembly/GCA_002797635]
286. *Actinomycetospora chiangmaiensis*	NCBI	GCA_000379625.1_ASM37962v1 [https://www.ncbi.nlm.nih.gov/assembly/GCA_000379625]
287. *Pseudonocardia endophytica*	NCBI	GCA_004339565.1_ASM433956v1 [https://www.ncbi.nlm.nih.gov/assembly/GCA_004339565]
288. *Sphingomonas* sp. YZ-8	NCBI	GCA_003660165.1_ASM366016v1 [https://www.ncbi.nlm.nih.gov/assembly/GCA_003660165]
289. *Pseudonocardia autotrophica*	NCBI	GCA_002119215.1_ASM211921v1 [https://www.ncbi.nlm.nih.gov/assembly/GCA_002119215]
290. *Rhodococcus* sp. S2-17	NCBI	GCA_003130705.1_ASM313070v1 [https://www.ncbi.nlm.nih.gov/assembly/GCA_003130705]
291. Nocardia niigatensis	NCBI	GCA_000308655.1_ASM30865v1 [https://www.ncbi.nlm.nih.gov/assembly/GCA_000308655]
292. Dactylosporangium aurantiacum	NCBI	GCA_000716715.1_ASM71671v1 [https://www.ncbi.nlm.nih.gov/assembly/GCA_000716715]
293. Allokutzneria albata	NCBI	GCA_900103775.1_IMG-taxon_2634166198 [https://www.ncbi.nlm.nih.gov/assembly/GCA_900103775]
294. Streptomyces tsukubensis	NCBI	GCA_002007125.1_ASM200712v1 [https://www.ncbi.nlm.nih.gov/assembly/GCA_002007125]
295. *Nocardia seriolae*	NCBI	GCA_001865855.1_ASM186585v1 [https://www.ncbi.nlm.nih.gov/assembly/GCA_001865855]
296. Actinobacteria bacterium	NCBI	GCA_004376625.1_ASM437662v1 [https://www.ncbi.nlm.nih.gov/assembly/GCA_004376625]
297. Enhydrobacter aerosaccus SK60	NCBI	GCA_000175915.1_ASM17591v1 [https://www.ncbi.nlm.nih.gov/assembly/GCA_000175915]
298. Amycolatopsis sp. BJA-103	NCBI	GCA_002849735.1_ASM284973v1 [https://www.ncbi.nlm.nih.gov/assembly/GCA_002849735]

Table **S3**: Secondary Age Calibrations for Species and Gene Trees

Phylogeny	Node	Upper limit (Ga)	Lower limit (Ga)
Species Tree	Root	4.400	-1
	Crown Chloroflexi	3.374	2.674
	Stem Chloroflexia	2.782	2.053
	Crown Chloroflexia	2.041	1.645
	Common Ancestor of Ktedonobacteria and Dehalococcoidia	2.995	1.793
	Crown Dehalococcoidia	2.013	0.789
	Common Ancestor of Anaerolineae and Chloroflexia	3.124	2.384
BVMOs Gene Tree	Root	3.200	1.200
	Common Ancestor of *SAR202* Io17-Chloro-G1 and Casp-Chloro-G4	1.623	0.988
	Common Ancestor of *SAR202* Io17-Chloro-G6 and Io17-Chloro-G8	0.620	0.358
	Common Ancestor of *SAR202* Io17-Chloro-G2 and Io17-Chloro-G9	0.587	0.265
	Common Ancestor of *SAR202* Io17-Chloro-G7 and Casp-Chloro-G2	1.083	0.721
	Common Ancestor of *SAR202* Io17-Chloro-G6 and Casp-Chloro-G3	1.343	0.891

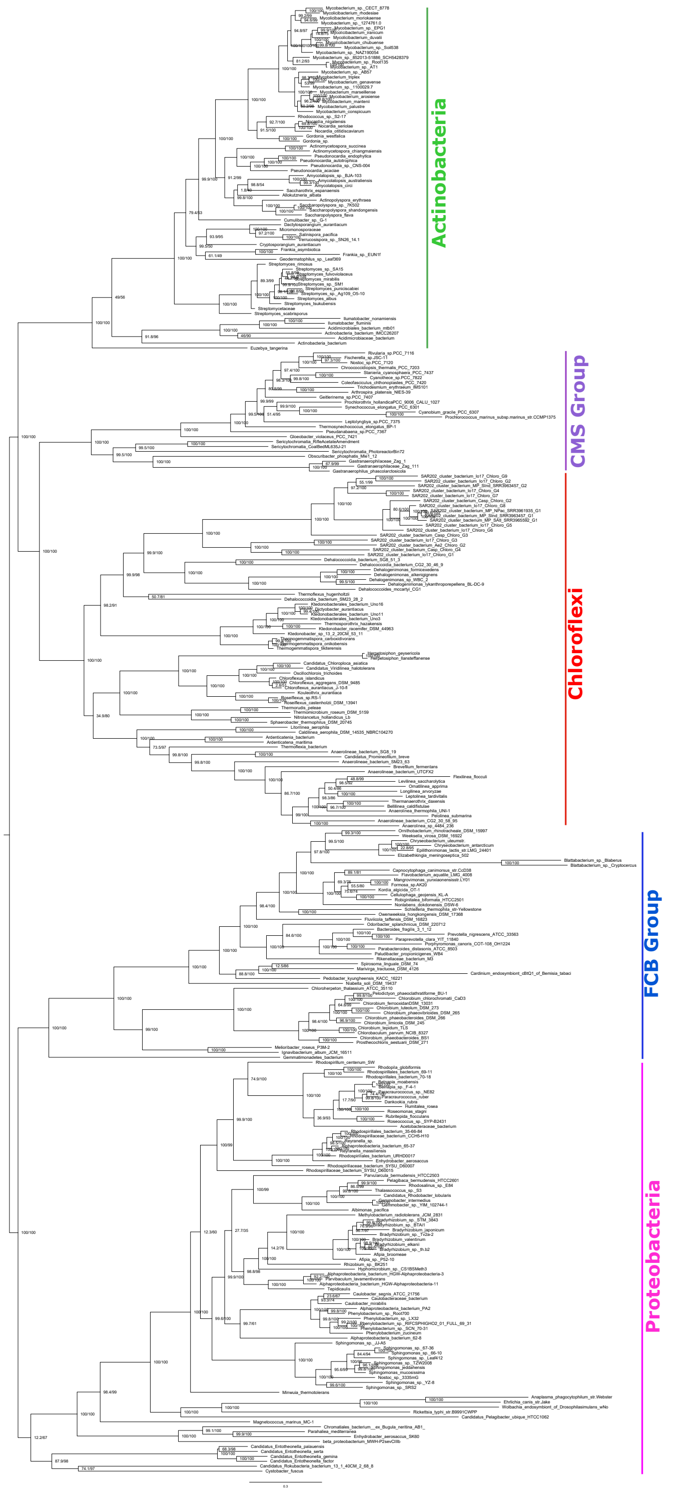

Figure **S1**: Rooted maximum likelihood species tree of BVMO-containing taxa, including the SAR202 cluster within Chloroflexi. Bipartitions are labeled with percentage support values (approximate likelihood ratio/bootstrap). This tree is manually rooted so that Actinobacteria, Chloroflexi and the CMS (Cyanobacteria-Melainabacteria-Sericytochromatia) group are in one clade while Proteobacteria and the FCB (Fibrobacteres-Chlorobi-Bacteroidete) group are in the other clade (as discussed in the Methods section in the main text).

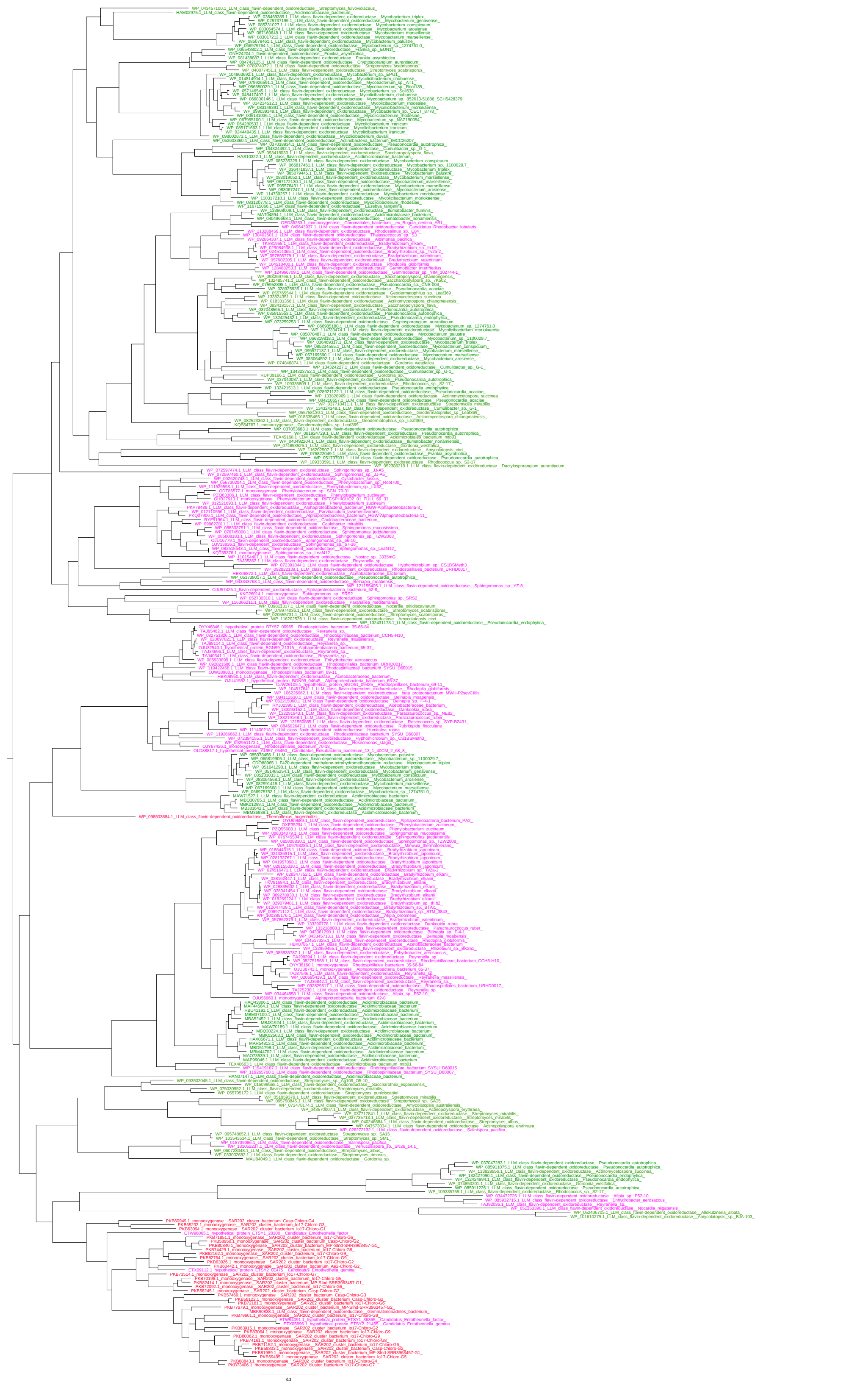

Figure **S2**: Rooted maximum likelihood tree of BVMO homologs. Bipartitions are labeled with percentage support values (approximate likelihood ratio/bootstrap). This tree is rooted using the minimal ancestor deviation (MAD) method [2] (as discussed in the Methods section in the main text). The taxa names of Chloroflexi, Actinobacteria, and Proteobacteria in the tips of this gene tree are colored in red, green, and neon pink, respectively.

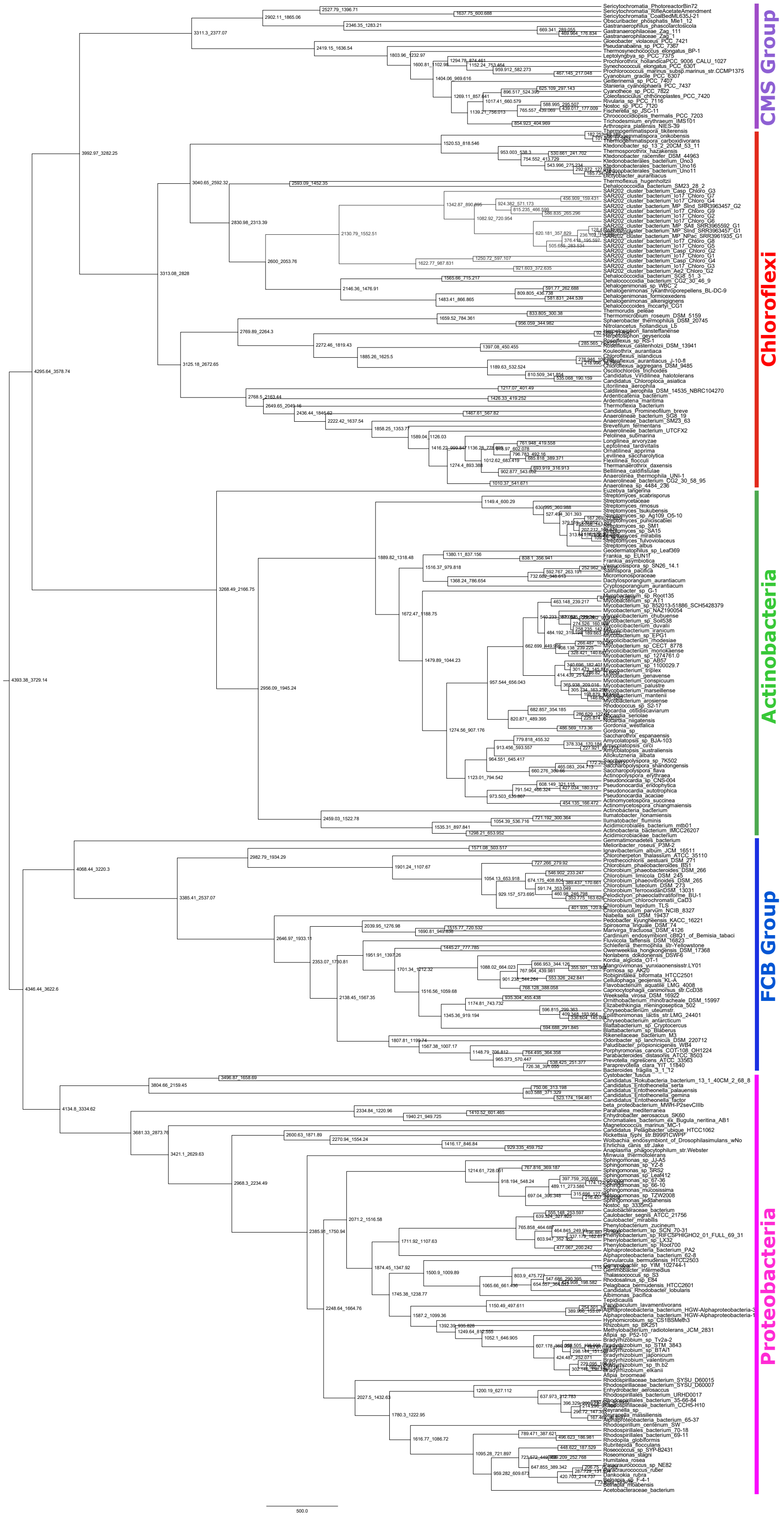

Figure **S3**: Species tree chronogram of BVMO-containing taxa, including the SAR202 cluster within Chloroflexi. Values on nodes represent 95% confidence intervals of inferred posterior age distributions.

Table **S4**: The 95% Confidence Intervals for Prior and Posterior Age Values of Representative Nodes on Species Tree

Node	Prior	Posterior
Root of Species Tree	4.382 Ga – 3.701 Ga	4.393 Ga – 3.729 Ga
Crown Chloroflexi	3.364 Ga – 2.848 Ga	3.313 Ga – 2.828 Ga
Stem Chloroflexia	2.751 Ga – 2.234 Ga	2.770 Ga – 2.264 Ga
Crown Chloroflexia	1.888 Ga – 1.629 Ga	1.885 Ga – 1.626 Ga
Common Ancestor of Classes Ktedonobacteria and Dehalococcoidia	3.052 Ga – 2.599 Ga	3.041 Ga – 2.592 Ga
Crown Dehalococcoidia	1.559 Ga – 0.949 Ga	1.483 Ga – 0.867 Ga
Common Ancestor of Classes Anaerolineae and Chloroflexia	3.129 Ga – 2.671 Ga	3.125 Ga – 2.672 Ga

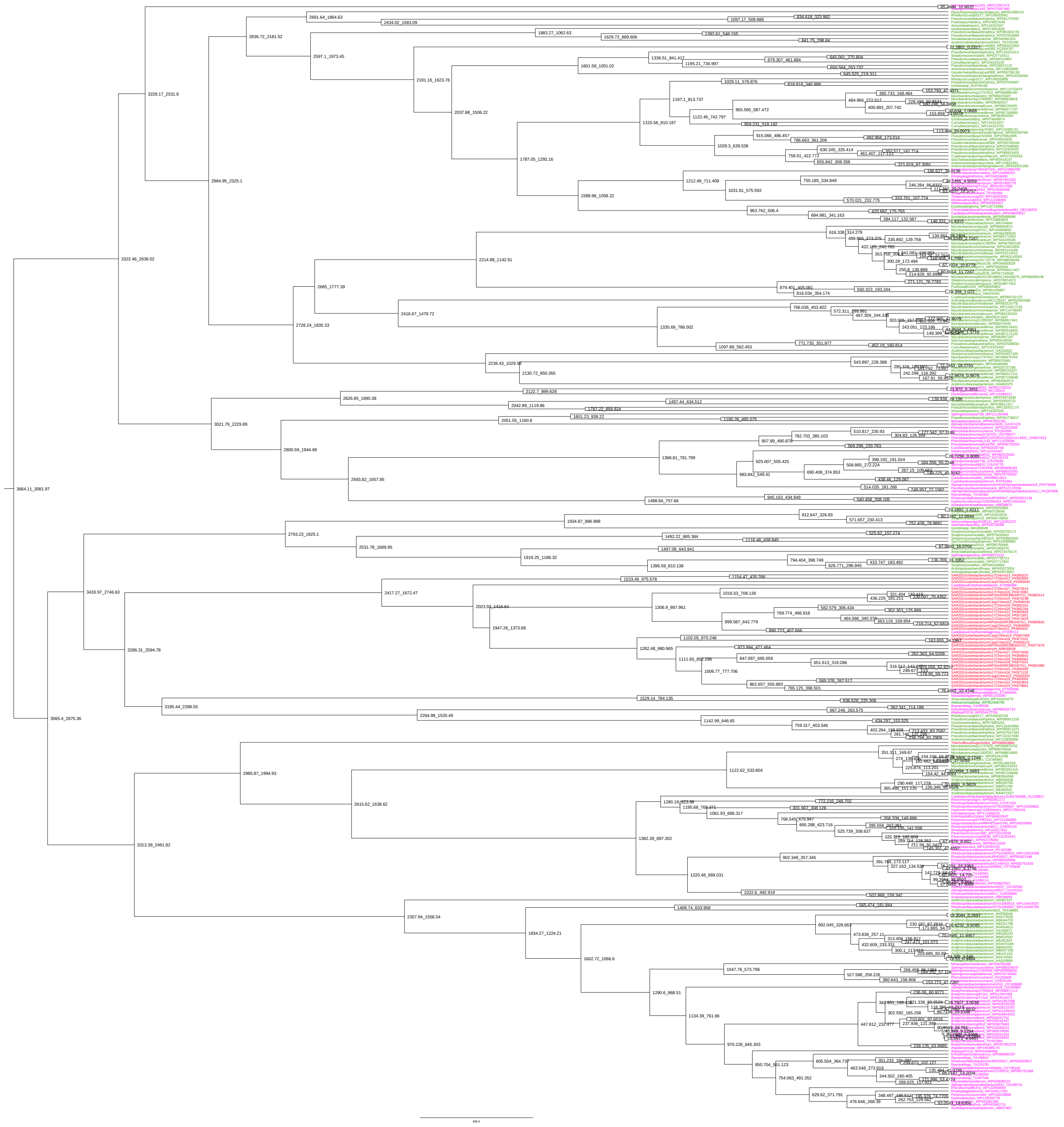

Figure **S4**: Gene tree chronogram of BVMO homologs. Values on nodes represent 95% confidence intervals of inferred posterior age distributions. The taxa names of Chloroflexi, Actinobacteria, and Proteobacteria in the tips of this gene tree chronogram are colored in red, green, and neon pink, respectively.

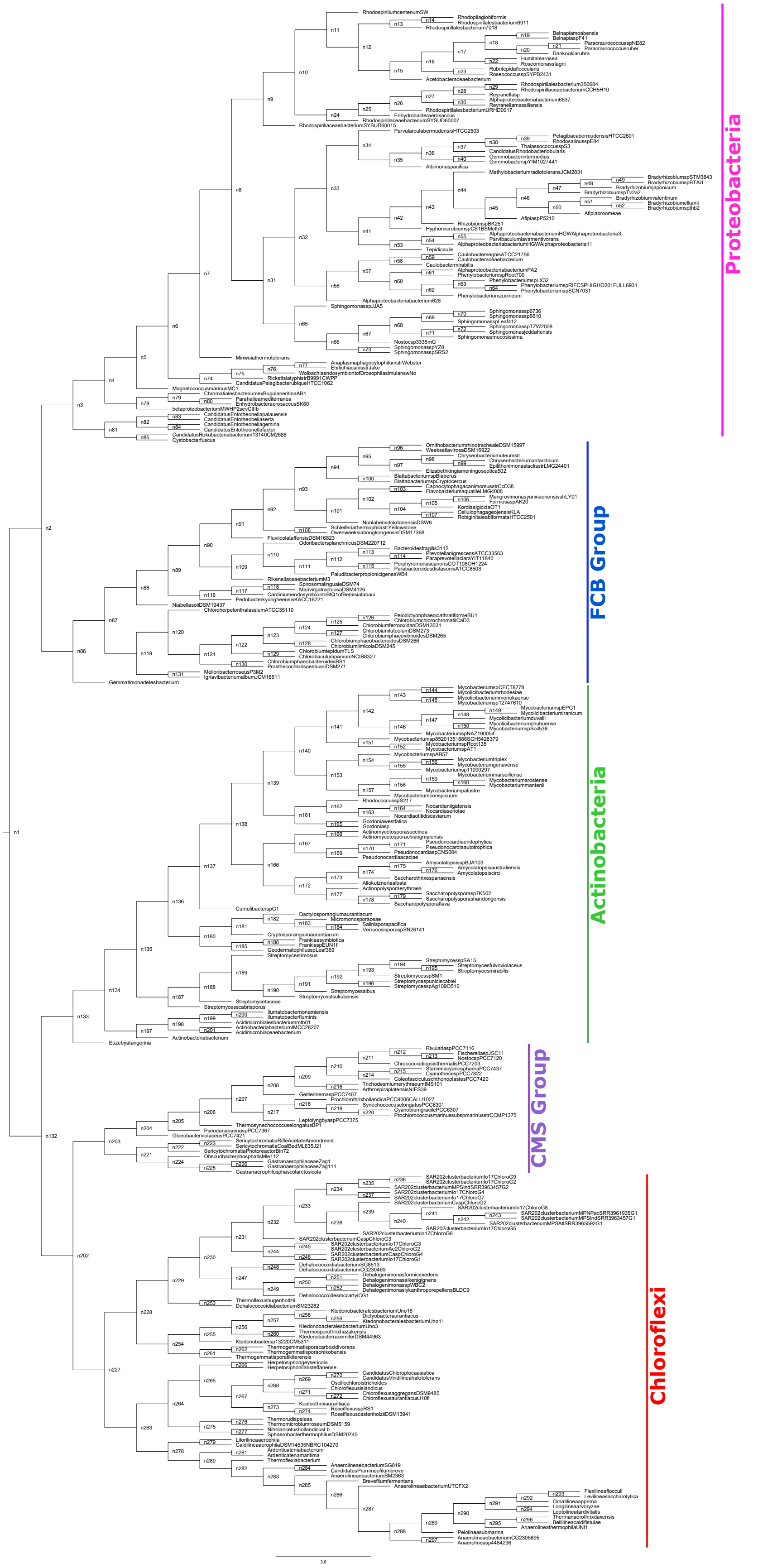

Figure **S5**: Rooted species tree cladogram with RANGER-DTL nodes mapped (n#).

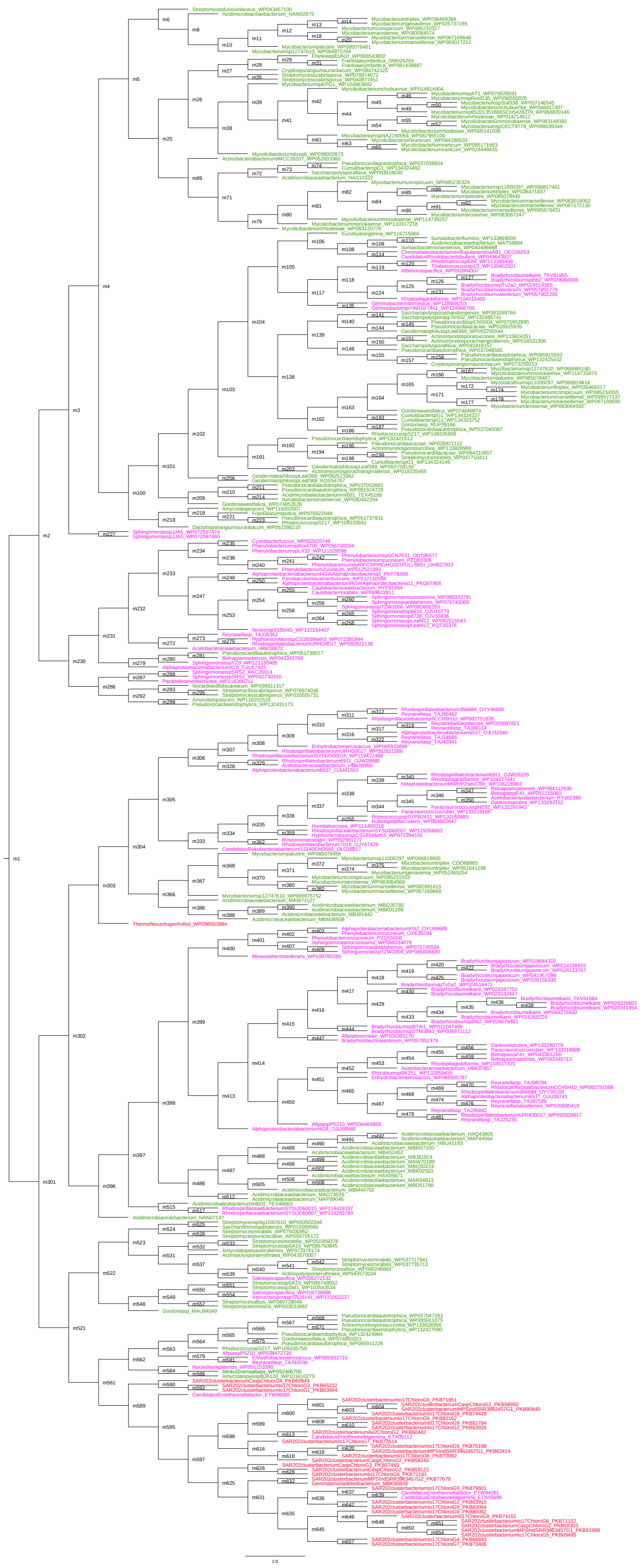

Figure **S6**: Rooted BVMO homolog tree cladogram with RANGER-DTL nodes mapped (m#). The taxa names of Chloroflexi, Actinobacteria, and Proteobacteria in the tips of this gene tree cladogram are colored in red, green, and neon pink, respectively.

Table **S5**: Weighted Means and 95% CIs of the Older and Younger Bounds for HGT Events

(Refer to Figure **S5** and Figure **S6** for the information of node labels ”n#” and ”m#”.)

HGT ID	Node in Gene Tree	Recipient Branch in Species Tree (older bound, younger bound)	Recipient Weights	Weighted Mean Ages (Ma) (older bound, younger bound)	95% CI (Ma) (older bound, younger bound)
1	m561	(n230, n231)	100 %	(2350.04, 1830.44)	(2598.23 - 2056.45, 2110.15 - 1535.13)
2	m3	(n134, n135)	23 %	(2002.70, 1286.83)	(2407.04 - 1616.46, 1552.85 - 1054.73)
		(n136, n180)	11 %		
		(n197, n198)	66 %		
3	m398	(n32, n56)	100 %	(1595.38, 1421.68)	(1847.61 - 1341.72, 1703.12 - 1130.51)
4	m397	(n32, n56)	100 %	(1595.38, 1421.68)	(1847.61 - 1341.72, 1703.12 - 1130.51)
5	m101	(n135, n136)	73 %	(1465.22, 1268.09)	(1722.05 - 1252.46, 1471.50 - 1078.70)
		(n138, n166)	13 %		
		(n198, n199)	14 %		
6	m450	(n9, n10)	65 %	(1426.47, 1148.75)	(1659.50 - 1203.30, 1363.03 - 955.47)
		(n12, n15)	10 %		
		(n24, n25)	25 %		
7	m27	(n136, n180)	100 %	(1420.51, 1252.02)	(1667.97 - 1201.51, 1506.32 - 984.37)
8	m209	(n198, n199)	100 %	(1188.4, 770.51)	(1570.97 - 892.80, 1068.74 - 583.46)
9	m210	(n198, n199)	100 %	(1188.4, 770.51)	(1570.97 - 892.80, 1068.74 - 583.46)
10	m118	(n34, n35)	87 %	(1106.39, 761.17)	(1320.90 - 907.90, 942.82 - 603.34)
		(n50, n51)	13 %		
11	m452	(n12, n15)	100 %	(892.13, 764.41)	(1086.88 - 723.66, 957.47 - 608.02)
12	m563	(n24, n25)	100 %	(889.7, 440.02)	(1199.50 - 645.63, 645.03 - 317.33)
13	m579	(n24, n25)	100 %	(889.7, 440.02)	(1199.50 - 645.63, 645.03 - 317.33)
14	m626	(n233, n238)	100 %	(883.07, 473.44)	(1072.91 - 722.48, 617.93 - 358.03)
15	m25	(n147, n148)	38 %	(861.00, 719.55)	(1046.02 - 708.32, 898.87 - 566.69)
		(n136, n180)	19 %		
		(n198, n201)	43 %		
16	m413	(n44, n45)	100 %	(838.21, 472.71)	(1050.19 - 662.94, 600.62 - 355.46)
17	m584	(n172, n173)	100 %	(800.92, 748.67)	(967.57 - 648.89, 920.87 - 593.96)
18	m150	(n167, n168)	100 %	(797.08, 281.58)	(975.44 - 640.32, 448.32 - 161.99)
19	m144	(n167, n169)	100 %	(797.08, 619.41)	(975.44 - 640.32, 796.96 - 467.36)
20	m301	(n139, n161)	100 %	(794.51, 649.30)	(961.42 - 652.49, 818.11 - 485.25)
21	m104	(n35, n36)	76 %	(793.82, 588.95)	(985.13 - 629.09, 757.10 -452.10)
		(n36, n37)	24 %		
22	m108	(n199, n200)	100 %	(770.51, 418.98)	(1068.74 - 538.46, 728.29)
23	m584	(n252, n253)	100 %	(735.50, 624.62)	(924.00 - 568.82, 807.58 - 466.02)
24	m339	(n13, n14)	100 %	(574.24, 310.48)	(789.73 - 388.53, 493.71 - 185.01)

Continued

HGT ID	Node in Gene Tree	Recipient Branch in Species Tree (older bound, younger bound)	Recipient Weights	Weighted Mean Ages (Ma) (older bound, younger bound)	95% CI (Ma) (older bound, younger bound)
25	m254	(n57, n58)	50 %	(562.78, 419.13)	(687.26 - 453.97, 532.49 - 329.50)
		(n67, n68)	50 %		
26	m637	(n82, n84)	100 %	(562.30, 336.65)	(845.71 - 369.75, 561.40 - 197.73)
27	m645	(n234, n237)	90 %	(558.50, 279.98)	(688.41 - 446.07, 445.74 - 167.27)
		(n239, n240)	10 %		
28	m333	(n12, n13)	5 %	(552.2, 377.67)	(686.45 - 442.86, 501.16 - 274.61)
		(n17, n22)	95 %		
29	m232	(n25, n26)	84 %	(496.72, 317.45)	(672.75 - 385.20, 418.62 - 248.31)
		(n56, n57)	4 %		
		(n57, n60)	2 %		
		(n66, n67)	10 %		
30	m336	(n13, n14)	7 %	(470.75, 255.35)	(581.22 - 377.09, 357.08 - 176.56)
		(n20, n21)	28 %		
		(n16, n23)	65 %		
31	m140	(n167, n169)	31 %	(466.12, 256.36)	(596.10 - 364.73, 329.92 - 197.61)
		(n178, n179)	69 %		
32	m337	(n13, n14)	17 %	(460.79, 251.41)	(566.36 - 368.23, 343.86 - 179.92)
		(n13, n14)	30 %		
		(n13, n14)	53 %		
33	m564	(n170, n171)	100 %	(445.86, 286.04)	(601.87 - 323.96, 419.72 - 178.85)
34	m157	(n170, n171)	100 %	(445.86, 286.04)	(601.87 - 323.96, 419.72 - 178.85)
35	m272	(n25, n26)	100 %	(440.02, 282.48)	(645.03 - 317.33, 394.78 - 205.80)
36	m247	(n54, n55)	89 %	(423.82, 194.93)	(537.54 - 321.19, 295.40 - 131.38)
		(n66, n67)	11 %		
37	m550	(n183, n184)	100 %	(402.76, 150.48)	(606.92 - 263.68, 253.39 - 87.56)
38	m41	(n143, n144)	32 %	(401.49 , 292.86)	(491.68 - 329.52, 384.50 - 210.74)
		(n141, n151)	68 %		
39	m71	(n142, n143)	100 %	(394.41, 315.27)	(483.9 - 314.88, 405.16 - 235.75)
40	m646	(n239, n240)	100 %	(381.51, 274.13)	(505.97 - 281.41, 376.26 - 195.07)
41	m619	(n239, n240)	100 %	(381.51, 274.13)	(505.97 - 281.41, 376.26 - 195.07)
42	m44	(n143, n144)	50 %	(378.21, 261.97)	(467.66 - 307.59, 338.86 - 192.30)
		(n141, n151)	50 %		
43	m42	(n143, n144)	45 %	(372.12 , 253.89)	(460.37 - 301.44, 329.70 - 184.75)
		(n141, n151)	55 %		
44	m117	(n36, n40)	63 %	(371.07, 123.28)	(452.61 - 294.37, 167.32 - 86.79)
		(n50, n51)	37 %		
45	m401	(n68, n71)	100 %	(365.38, 206.57)	(482.37 - 277.32, 311.30 - 124.05)

Continued

HGT ID	Node in Gene Tree	Recipient Branch in Species Tree (older bound, younger bound)	Recipient Weights	Weighted Mean Ages (Ma) (older bound, younger bound)	95% CI (Ma) (older bound, younger bound)
46	m26	(n147, n148)	88 %	(361.39, 326.84)	(430.74 - 299.20, 397.26 - 265.15)
		(n136, n180)	12 %		
47	m81	(n153, n157)	100 %	(323.05, 280.01)	(408.56 - 252.13, 362.58 - 209.57)
48	m338	(n13, n14)	32 %	(317.05, 186.08)	(406.19 - 233.81, 261.19 - 125.20)
		(n20, n21)	68 %		
49	m166	(n143, n145)	100 %	(315.27, 225.34)	(405.16 - 235.75, 333.62 - 141.45)
50	m345	(n18, n19)	100 %	(302.11, 40.09)	(413.53 - 214.94, 74.22 - 20.40)
51	m548	(n190, n191)	100 %	(286.33, 234.65)	(380.13 - 219.53, 305.71 - 179.41)
52	m304	(n157, n158)	100 %	(280.01, 224.98)	(362.58 - 209.57, 306.73 - 164.28)
53	m10	(n157, n158)	100 %	(280.01, 224.98)	(362.58 - 209.57, 306.73 - 164.28)
54	m164	(n157, n158)	100 %	(280.01, 224.98)	(362.58 - 209.57, 306.73 - 164.28)
55	m8	(n157, n158)	100 %	(280.01, 224.98)	(362.58 - 209.57, 306.73 - 164.28)
56	m85	(n154, n155)	100 %	(253.41, 215.05)	(336.94 - 183.83, 300.24 - 149.42)
57	m370	(n154, n155)	100 %	(253.41, 215.05)	(336.95 - 183.83, 300.24 -149.42)
58	m371	(n154, n155)	100 %	(253.41, 215.05)	(336.95 - 183.83, 300.24 -149.42)
59	m171	(n154, n155)	100 %	(253.41, 215.05)	(336.95 - 183.83, 300.24 - 149.42)
60	m240	(n63, n64)	100 %	(236.98, 122.65)	(335.45 - 161.76, 207.89 - 74.34)
61	m522	(n191, n192)	100 %	(234.65, 187.77)	(305.71 - 179.41, 247.87 - 142.50)
62	m124	(n50, n51)	100 %	(220.84, 159.53)	(302.13 - 159.01, 226.52 - 106.21)
63	m13	(n155, n156)	100 %	(215.05, 120.26)	(300.24 - 149.42, 190.65 - 72.60)
64	m12	(n155, n156)	100 %	(215.05, 120.26)	(300.24 - 149.42, 190.65 - 72.60)
65	m316	(n27, n30)	100 %	(210.31, 94.52)	(297.65 - 148.08, 170.15 - 47.31)
66	m309	(n27, n28)	100 %	(210.31, 145.90)	(297.65 - 148.08, 216.72 - 98.91)
67	m49	(n147, n150)	100 %	(210.09, 146.05)	(273.32 - 156.01, 204.35 - 97.78)
68	m417	(n51, n52)	100 %	(159.5, 102.61)	(226.52 - 106.21, 161.07 - 61.26)

Table **S6**: Direction of HGT Events

HGT ID	Phylum of HGT Donor	Phylum of HGT Recipient
1	Actinobacteria	Chloroflexi
2	Proteobacteria	Actinobacteria
3	Actinobacteria	Proteobacteria
4	Proteobacteria	Proteobacteria
5	Actinobacteria	Actinobacteria
6	Proteobacteria	Proteobacteria
7	Actinobacteria	Actinobacteria
8	Actinobacteria	Actinobacteria
9	Actinobacteria	Actinobacteria
10	Proteobacteria	Proteobacteria
11	Proteobacteria	Proteobacteria
12	Actinobacteria	Proteobacteria
13	Proteobacteria	Proteobacteria
14	Chloroflexi	Chloroflexi
15	Actinobacteria	Actinobacteria
16	Proteobacteria	Proteobacteria
17	Actinobacteri	Actinobacteria
18	Actinobacteria	Actinobacteria
19	Actinobacteria	Actinobacteria
20	Proteobacteria	Actinobacteria
21	Actinobacteria	Proteobacteria
22	Proteobacteria	Actinobacteria
23	Actinobacteria	Actinobacteria
24	Proteobacteria	Proteobacteria
25	Proteobacteria	Proteobacteria
26	Chloroflexi	Proteobacteria
27	Chloroflexi	Chloroflexi
28	Proteobacteria	Proteobacteria
29	Proteobacteria	Proteobacteria
30	Proteobacteria	Proteobacteria
31	Actinobacteria	Actinobacteria
32	Proteobacteria	Proteobacteria
33	Actinobacteria	Actinobacteria
34	Actinobacteria	Actinobacteria
35	Proteobacteria	Proteobacteria
36	Proteobacteria	Proteobacteria
37	Actinobacteria	Actinobacteria
38	Actinobacteria	Actinobacteria
39	Actinobacteria	Actinobacteria
40	Chloroflexi	Chloroflexi
41	Chloroflexi	Chloroflexi
42	Actinobacteria	Actinobacteria
43	Actinobacteria	Actinobacteria
44	Proteobacteria	Proteobacteria
45	Proteobacteria	Proteobacteria
46	Actinobacteria	Actinobacteria
47	Actinobacteria	Actinobacteria
48	Proteobacteria	Proteobacteria

Continued

HGT ID	Phylum of HGT Donor	Phylum of HGT Recipient
49	Actinobacteria	Actinobacteria
50	Proteobacteria	Proteobacteria
51	Actinobacteria	Actinobacteria
52	Proteobacteria	Actinobacteria
53	Actinobacteria	Actinobacteria
54	Actinobacteria	Actinobacteria
55	Actinobacteria	Actinobacteria
56	Actinobacteria	Actinobacteria
57	Actinobacteria	Actinobacteria
58	Actinobacteria	Actinobacteria
59	Actinobacteria	Actinobacteria
60	Proteobacteria	Proteobacteria
61	Actinobacteria	Actinobacteria
62	Proteobacteria	Proteobacteria
63	Actinobacteria	Actinobacteria
64	Actinobacteria	Actinobacteria
65	Proteobacteria	Proteobacteria
66	Proteobacteria	Proteobacteria
67	Actinobacteria	Actinobacteria
68	Proteobacteria	Proteobacteria

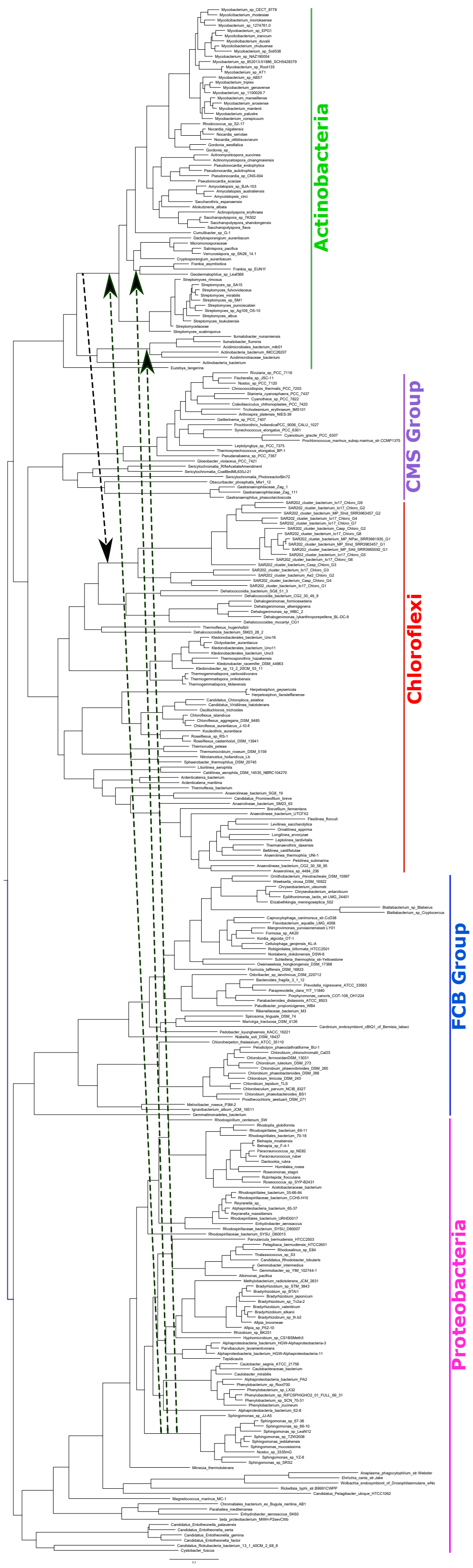

Figure **S7**: HGT events whose older bounds are older than 2000 Ma. The HGT event from Actinobacteria to Chloroflexi is HGT #1 in Table **S5**. The HGT events from Proteobacteria to Actinobacteria are HGT #2 in Table **S5**.

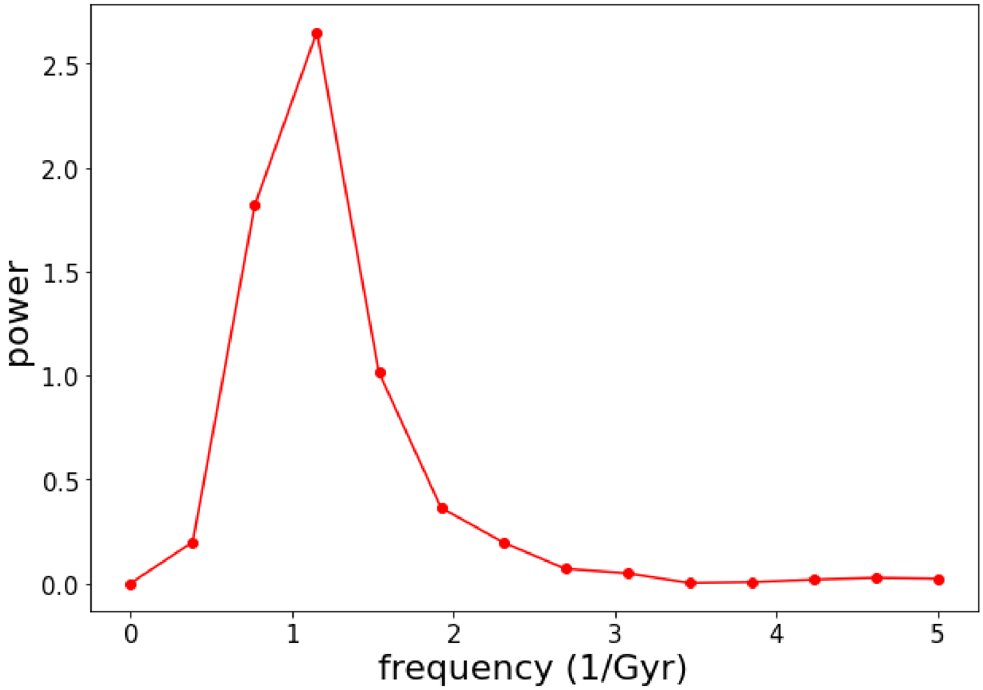

Figure **S8**: The power spectrum for the fluctuations x_i [equation (12) in the Methods section], using the SAR202 BVMO diversification rates. The probability that a randomly branching gene tree would yield a spectrum with such a sharp peak is $p < 0.01$.

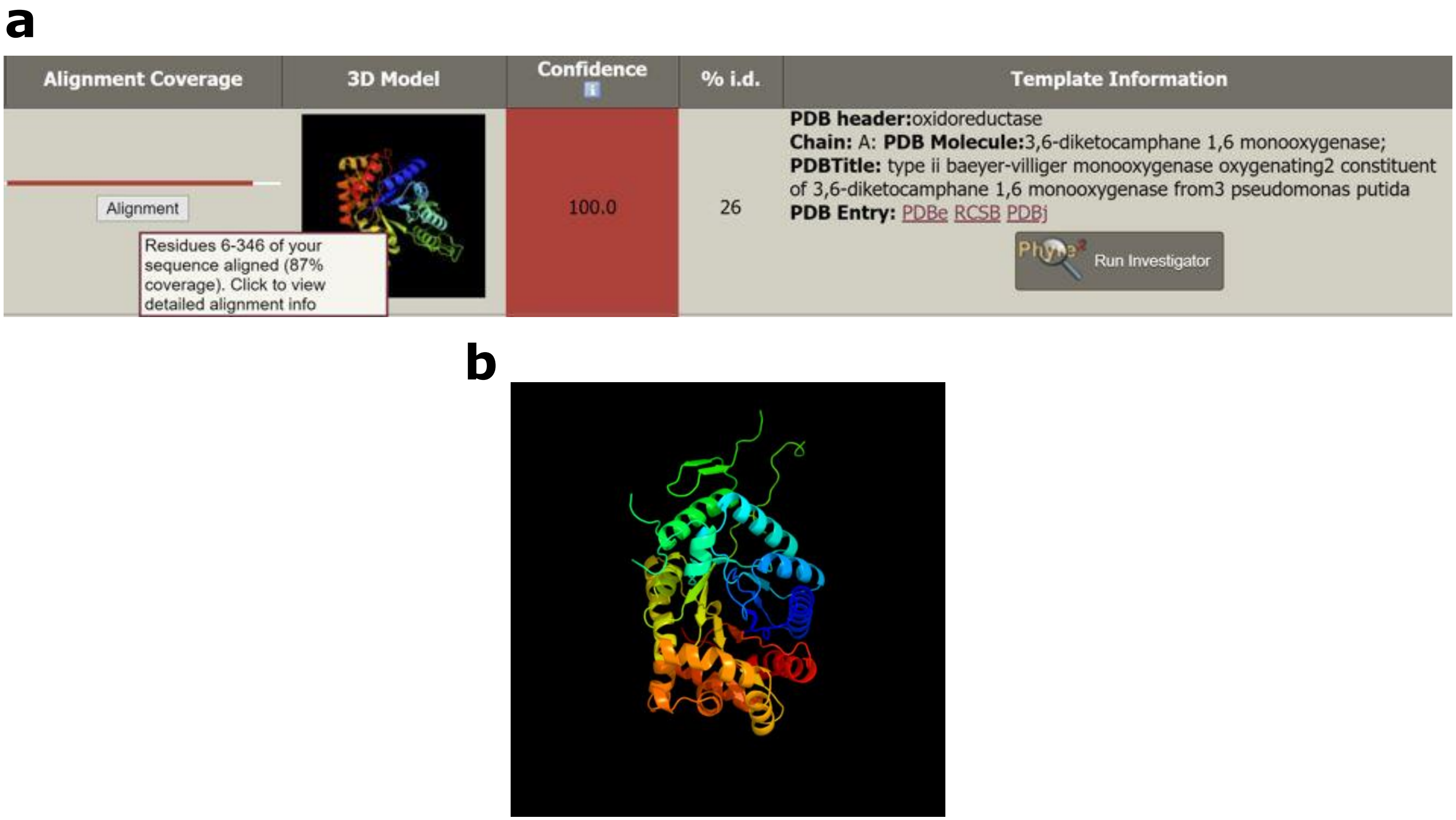

Figure **S9**: (a) A Phyre model [3] of BVMO. The confidence and coverage in the model are 100% and 87%, respectively. This means that 87% of the sequence has been modelled with 100% confidence by the template of a Type II Baeyer-Villiger monooxygenase. (b) A three-dimensional model of the Type II Baeyer-Villiger monooxygenase template.

References

[1] Soo, R. M., Hemp, J., Parks, D. H., Fischer, W. W. & Hugenholtz, P. On the origins of oxygenic photosynthesis and aerobic respiration in Cyanobacteria. *Science* **355**, 1436–1440 (2017).

[2] Tria, F. D. K., Landan, G. & Dagan, T. Phylogenetic rooting using minimal ancestor deviation. *Nature Ecology & Evolution* **1**, 1–7 (2017).

[3] Kelley, L. A., Mezulis, S., Yates, C. M., Wass, M. N. & Sternberg, M. J. The Phyre2 web portal for protein modeling, prediction and analysis. *Nature Protocols* **10**, 845 (2015).
