# Supplementary material for: Oxidative metabolisms catalyzed Earth’s oxygenation
Source: Nat Commun. 2022 Mar 14;13:1328. doi: 10.1038/s41467-022-28996-0 (PMC8921266; doi:10.1038/s41467-022-28996-0)
Supplement: Supplementary file 3 — Description of Additional Supplementary Files [file 41467_2022_28996_MOESM3_ESM.pdf]

Description of Additional Supplementary Files

File Name: Supplementary Data 1
Description: Aligned Sequences for Species Tree.fasta

File Name: Supplementary Data 2
Description: Aligned Sequences for Gene Tree.fasta

File Name: Supplementary Data 3
Description: Rooted Species Tree.treefile

File Name: Supplementary Data 4
Description: Rooted Gene Tree.treefile

File Name: Supplementary Data 5
Description: Rooted Species Tree.chronogram

File Name: Supplementary Data 6
Description: Rooted Gene Tree.chronogram
