# Supplementary material for: Oxidative metabolisms catalyzed Earth’s oxygenation
Source: Nat Commun. 2022 Mar 14;13:1328. doi: 10.1038/s41467-022-28996-0 (PMC8921266; doi:10.1038/s41467-022-28996-0)
Supplement: Supplementary file 5 — Reporting Summary [file 41467_2022_28996_MOESM5_ESM.pdf]

nature portfolio

Corresponding author(s): Haitao Shang

Last updated by author(s): Jan 31, 2022

Reporting Summary

Nature Portfolio wishes to improve the reproducibility of the work that we publish. This form provides structure for consistency and transparency in reporting. For further information on Nature Portfolio policies, see our Editorial Policies and the Editorial Policy Checklist.

Statistics

For all statistical analyses, confirm that the following items are present in the figure legend, table legend, main text, or Methods section.

n/a	Confirmed	
☐	☒	The exact sample size (*n*) for each experimental group/condition, given as a discrete number and unit of measurement
☒	☐	A statement on whether measurements were taken from distinct samples or whether the same sample was measured repeatedly
☐	☒	The statistical test(s) used AND whether they are one- or two-sided<br>*Only common tests should be described solely by name; describe more complex techniques in the Methods section.*
☒	☐	A description of all covariates tested
☐	☒	A description of any assumptions or corrections, such as tests of normality and adjustment for multiple comparisons
☐	☒	A full description of the statistical parameters including central tendency (e.g. means) or other basic estimates (e.g. regression coefficient) AND variation (e.g. standard deviation) or associated estimates of uncertainty (e.g. confidence intervals)
☐	☒	For null hypothesis testing, the test statistic (e.g. *F*, *t*, *r*) with confidence intervals, effect sizes, degrees of freedom and *P* value noted<br>*Give P values as exact values whenever suitable.*
☐	☒	For Bayesian analysis, information on the choice of priors and Markov chain Monte Carlo settings
☒	☐	For hierarchical and complex designs, identification of the appropriate level for tests and full reporting of outcomes
☒	☐	Estimates of effect sizes (e.g. Cohen's *d*, Pearson's *r*), indicating how they were calculated

Our web collection on statistics for biologists contains articles on many of the points above.

Software and code

Policy information about availability of computer code

Data collection	No software or code was used for data collection.
Data analysis	Custom code used in data analysis is available in a GitHub repository (https://github.com/Shang-Research/OxidativeMetabolisms). All third-party softwares used in this study are publicly available and listed as follows. Phyre2 structural homology recognition server (https://www.sbg.bio.ic.ac.uk/phyre2) was used to check the homology of the Baeyer-Villiger monooxygenases genes that were not well annotated in the original database. MAFFT (version 7.487) was used to align protein sequences. Clustal X (version 2.0) was used to visualize alignments and manually delete the poorly aligned regions. SequenceMatrix (version 1.8) was used to concatenate the aligned ribosomal protein sequences. IQ-TREE (version 1.6.3) with the ultrafast bootstrap algorithm was used to infer the maximum-likelihood species tree and gene tree. MAD (version 2.2) was used to infer the root position of the gene tree. PhyloBayes (version 4.1c) was used to run molecular clocks and estimate the divergence time of the species tree and gene tree. RANGER-DTL (version 2.0) was used to perform phylogenetic reconciliation and infer horizontal gene transfers.

For manuscripts utilizing custom algorithms or software that are central to the research but not yet described in published literature, software must be made available to editors and reviewers. We strongly encourage code deposition in a community repository (e.g. GitHub). See the Nature Portfolio guidelines for submitting code & software for further information.

Data

Policy information about availability of data

All manuscripts must include a data availability statement. This statement should provide the following information, where applicable:

- Accession codes, unique identifiers, or web links for publicly available datasets
- A description of any restrictions on data availability
- For clinical datasets or third party data, please ensure that the statement adheres to our policy

All data needed to evaluate the conclusions in the paper are presented in the paper and/or the Supplementary Information. All sequencing data used in this study are provided in Supplement Data 1 and 2. All trees generated in this study are provided in Supplement Data 3, 4, 5 and 6, and are also available on Github (https://github.com/Shang-Research/OxidativeMetabolisms). Source data are provided with this paper and are also available in public databases (PATRIC: https://www.patricbrc.org; IMG: https://img.jgi.doe.gov/; NCBI: https://www.ncbi.nlm.nih.gov). The information of databases, accession numbers, and hyperlinks for the sequence data used in this study are provided Table S2 in Supplementary Information.

Field-specific reporting

Please select the one below that is the best fit for your research. If you are not sure, read the appropriate sections before making your selection.

☐ Life sciences ☐ Behavioural & social sciences ☒ Ecological, evolutionary & environmental sciences

For a reference copy of the document with all sections, see nature.com/documents/nr-reporting-summary-flat.pdf

Ecological, evolutionary & environmental sciences study design

All studies must disclose on these points even when the disclosure is negative.

Study description	This study proposes an innovative mechanism to interpret Earth's oxygenation in deep time, reconstruct the evolutionary history of a key enzyme family responsible for this mechanism, and shows the temporal consistency of the diversification of this enzyme family with the Proterozoic and Phanerozoic atmospheric oxygenation.
Research sample	All data in this study are from existing databases. To reconstruct the species tree, we collected the sequences that are homologous to 30 ribosomal proteins (Table S1 in the Supplementary Information) of the SAR202 cluster bacteria. The ribosomal proteins are from 298 taxa (Table S2 in the Supplementary Information) in the public databases (PATRIC: https://www.patricbrc.org, IMG: https://img.jgi.doe.gov, and NCBI: https://www.ncbi.nlm.nih.gov). To reconstruct the gene tree of Baeyer-Villiger monooxygenases (BVMOs), 330 BVMO sequences were recovered from the NCBI database (https://www.ncbi.nlm.nih.gov) via a BLAST search with the BVMO sequence of SAR202 cluster bacteriumIo17-Chloro-G4 (NCBI Query ID: PKB68843.1) as a query. These ribosomal protein sequences and BVMO sequences were sampled and used in this work because SAR202 cluster bacteria are responsible for the core hypothesis and mechanisms suggested in this study.
Sampling strategy	Ribosomal protein sequences were identified for SAR202 cluster bacteria via protein BLAST of homologous sequences, and used to reconstruct the species tree in this study. The protein sequence data of Baeyer-Villiger monooxygenases (BVMOs) were recovered from the NCBI database (https://www.ncbi.nlm.nih.gov) via a BLAST search with the BVMO sequence of SAR202 cluster bacteriumIo17-Chloro-G4 (NCBI Query ID: PKB68843.1) as a query; the top 330 hits of the BVMO sequences, which showed clear homology based on sequence similarity, were selected. These were almost all of the homologous sequences of ribosomal proteins and BVMOs (relevant to the hypothesis and mechanisms we suggested in this study) that were available when we collected data from the databases (e.g., NCBI: https://www.ncbi.nlm.nih.gov). Therefore there was no need to predetermine the sample size. Moreover, the taxa whose BVMOs appear in the gene tree are all included in the species tree so that the detection of horizontal gene transfers could be performed.
Data collection	The data used in this study were downloaded by the corresponding author from existing databases, including PATRIC (https://www.patricbrc.org), IMG (https://img.jgi.doe.gov/), and NCBI (https://www.ncbi.nlm.nih.gov). No new data were collected/generated by the authors in this study.
Timing and spatial scale	The data of ribosomal proteins for the species tree reconstruction were collected/downloaded from three existing, global-scale databases (PATRIC: https://www.patricbrc.org, IMG: https://img.jgi.doe.gov, and NCBI: https://www.ncbi.nlm.nih.gov) on 13 April, 2019. The data of Baeyer-Villiger monooxygenases (BVMOs) for the gene tree reconstruction were collected/downloaded from the global-scale NCBI database (https://www.ncbi.nlm.nih.gov) on 20 May, 2019. These two datasets of ribosomal proteins and BVMOs were both collected by one-time downloading; there was no time gap in our data collection.
Data exclusions	No data was excluded from the analyses in our study.
Reproducibility	All results presented in this study are based on the analyses of the existing datasets rather than laboratory or field experiments, so the reproducibility of our results can be verified using the softwares, code, and datasets provided in the Supplementary Information and GitHub (https://github.com/Shang-Research/OxidativeMetabolisms). We also compiled our code and the reproduced figures in a GitHub repository (https://github.com/Shang-Research/OxidativeMetabolisms).
Randomization	This study does not include experimental groups.

Blinding	Blinding was not relevant to this study, because we analyzed the existing databases but did not perform laboratory or field experiments.

Did the study involve field work? ☐ Yes ☒ No

Reporting for specific materials, systems and methods

We require information from authors about some types of materials, experimental systems and methods used in many studies. Here, indicate whether each material, system or method listed is relevant to your study. If you are not sure if a list item applies to your research, read the appropriate section before selecting a response.

Materials & experimental systems

n/a	Involved in the study
☒	☐ Antibodies
☒	☐ Eukaryotic cell lines
☒	☐ Palaeontology and archaeology
☒	☐ Animals and other organisms
☒	☐ Human research participants
☒	☐ Clinical data
☒	☐ Dual use research of concern

Methods

n/a	Involved in the study
☒	☐ ChIP-seq
☒	☐ Flow cytometry
☒	☐ MRI-based neuroimaging
